# Effects of vibrational excitation on the F + H_2_O → HF + OH reaction: dissociative photodetachment of overtone-excited [F–H–OH]^–^ †
†Electronic supplementary information (ESI) available. See DOI: 10.1039/c7sc03364h



**DOI:** 10.1039/c7sc03364h

**Published:** 2017-09-25

**Authors:** Amelia W. Ray, Jianyi Ma, Rico Otto, Jun Li, Hua Guo, Robert E. Continetti

**Affiliations:** a Department of Chemistry and Biochemistry , University of California, San Diego , 9500 Gilman Drive , La Jolla , California 92093-0340 , USA . Email: rcontinetti@ucsd.edu; b Institute of Atomic and Molecular Physics , Sichuan University , Chengdu , Sichuan 610065 , China . Email: majianyi81@163.com; c School of Chemistry and Chemical Engineering , Chongqing University , Chongqing 401331 , China; d Department of Chemistry and Chemical Biology , University of New Mexico , Albuquerque , New Mexico 87131 , USA

## Abstract

The reaction F + H_2_O → HF + OH is a four-atom system that provides an important benchmark for reaction dynamics. Hydrogen atom transfer at the transition state for this reaction is expected to exhibit a strong dependence on reactant vibrational excitation. In the present study, the vibrational effects are examined by photodetachment of vibrationally excited F^–^(H_2_O) precursor anions using photoelectron-photofragment coincidence (PPC) spectroscopy and compared with full six-dimensional quantum dynamical calculations on *ab initio* potential energy surfaces. Prior to photodetachment at *hν*_UV_ = 4.80 eV, the overtone of the ionic hydrogen bond mode in the precursor F^–^(H_2_O), 2*ν*_IHB_ at 2885 cm^–1^, was excited using a tunable IR laser. Experiment and theory show that vibrational energy in the anion can be effectively carried away by the photoelectron upon a Franck–Condon photodetachment, and also show evidence for an increase of branching into the F + H_2_O reactant channel. The experimental results suggest a greater role for product rotational excitation than theory. Improved potential energy surfaces and longer wavepacket propagation times would be helpful to further examine the nature of the discrepancy.

## Abstract 2

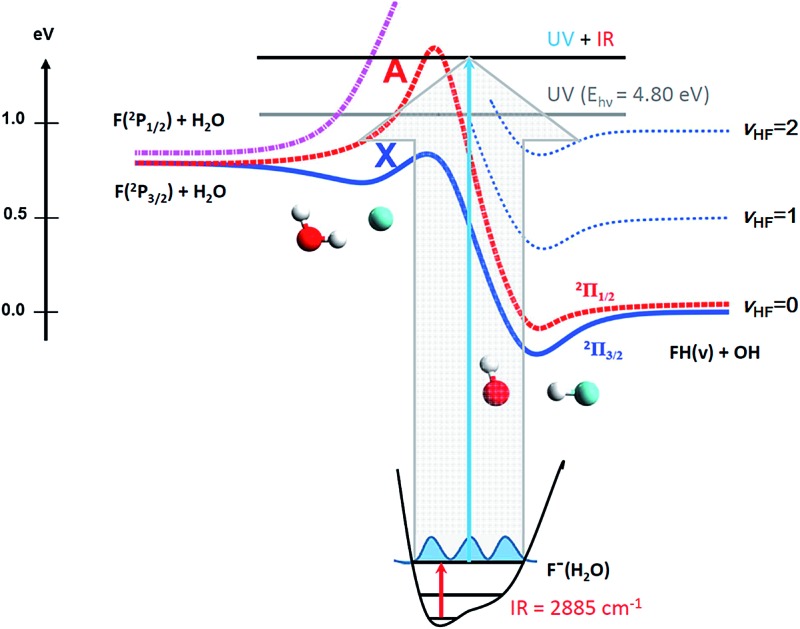
Photodetaching vibrationally excited FH_2_O^–^ channels energy into the reaction coordinate of the F + H_2_O reaction, as shown in this joint experimental-theoretical study.

## 1. Introduction

The effects of vibrational excitation on the rates and dynamics of chemical reactions in the gas phase are the focus of considerable interest. In the case of atom–diatom reactions, Polanyi's rules explain the differing effects of reactant vibration and translational energy as a function of reaction energetics,^1^ and recent work has extended these concepts toward polyatomic systems.^2–4^ Considerable experimental effort has been devoted toward exploration of mode-selective chemistry involving polyatomic molecules both in the gas phase^5–10^ and at surfaces.^11,12^ These studies indicate that excitation of vibrational modes with large displacements along a reaction coordinate can preferentially promote surmounting specific barriers, giving rise to mode-selective chemistry. The present work is a study of the effect of anion vibrational excitation on the dynamics of the neutral F + H_2_O → HF + OH reaction by means of direct IR absorption. This reaction is initiated near the transition state for the bimolecular reaction by photodetachment of F^–^(H_2_O).^13^ Excitation of a suitable mode in the precursor anion changes the Franck–Condon overlap with the neutral surface, and in favorable cases should provide considerable control over product energy disposal and dynamics. Here, we examine the effects of excitation of the overtone of the F–H–OH ionic hydrogen bond (IHB, 2*ν*_IHB_) in F^–^(H_2_O) using photoelectron-photofragment coincidence (PPC) spectroscopy. This anion mode is particularly interesting because it is well aligned with the neutral reaction coordinate and the stretching vibration of the H_2_O reactant. The experiment was carried out by coupling an infrared laser system into the ion beam line of the PPC spectrometer.^14^ The experimental results are compared to extensive full-dimensional (6-D) quantum dynamics calculations using *ab initio* anionic and neutral potential energy surfaces (PESs).

Neutralization of ionic species provides a useful approach to examining neutral reaction dynamics. Photodetachment photoelectron spectroscopy of negative ions has been used as a spectroscopy of the neutral transition state in isomerization and chemical reactions.^15–19^ These experiments probe neutral configurations as determined by the ground state anion precursor. To go beyond this restriction, Lineberger and coworkers, using negative ion–neutral–positive ion (NeNuPo) charge reversal spectroscopy, probed a broad portion of the neutral PES of Ag_3_ by photodetachment of Ag_3_^–^ followed by femtosecond photoionization to probe the time evolution of the neutral wavepacket.^20^ An alternative approach to sampling other configurations on the neutral surface is to vibrationally excite the anion precursor prior to photodetachment. Neumark and coworkers have demonstrated this approach by using stimulated Raman excitation of C_2_^–^ as demonstrated by photoelectron spectroscopy.^21,22^ However, stimulated Raman pumping is limited to those anion systems with optically accessible excited states below the photodetachment threshold. Other approaches including stimulated Raman-adiabatic passage (STIRAP) may also be applied on systems that have been extensively spectroscopically characterized.^23^ Lacking such information in the case of F^–^(H_2_O) we have taken the more general approach of direct absorption of infrared radiation used in a number of crossed molecular beam studies with vibrationally excited reactants^7,8^ as well as anionic cluster vibrational predissociation and vibrationally mediated photodetachment.^24^

**Fig. 1:**
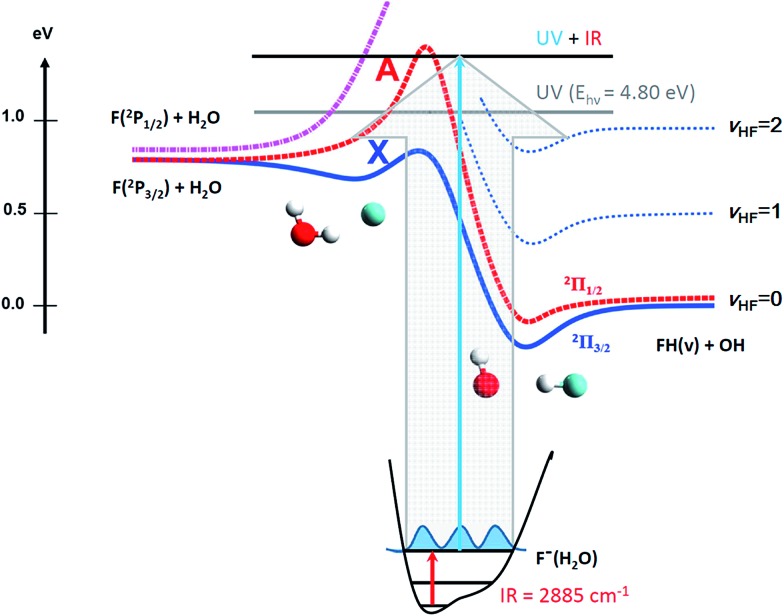
Potential energy diagram for the ground (X) and excited (A) states of the F + H_2_O → HF + OH reaction and a schematic 1D potential energy slice along the ionic hydrogen bond (IHB) of the F^–^(H_2_O). Anion and neutral surfaces not necessarily to scale.

We have previously studied the prototypical hydrogen-abstraction reaction F + H_2_O → FH_2_O* → HF + OH in a joint experimental/theoretical effort combining PPC spectroscopy with full six-dimensional (6D) quantum-dynamics simulations of the dissociative photodetachment process on *ab initio* PESs.^13^ This reaction provides an excellent case for studying the effects of vibrational excitation on the decay of neutral complexes near the transition state for a bimolecular reaction. The system is characterized by three low-lying PESs, shown schematically in Fig. 1. Asymptotically, the ground state F(^2^P_3/2_) correlates adiabatically to HF + OH(^2^Π_3/2_) along the ground (X) state and also accesses the higher-lying excited (A) state, leading to formation of spin-orbit-excited HF + OH(^2^Π_1/2_).^25–28^ The third surface corresponds to the endothermic reaction of spin-orbit-excited F(^2^P_1/2_) + H_2_O to adiabatically form electronically excited OH(^2^Σ^+^) + HF products. Reaction barriers separate reactant-channel and product-channel complexes on both the X and A states,^29–31^ and the pre-reactive F–H_2_O complex was recently shown to possibly exert a strong influence on the overall reactivity.^32^

Reaction rate measurements on this system suggested a tunneling-mediated reaction at low temperatures, based in part on the observed kinetic-isotope effect and the observation of no temperature dependence.^33^ Wang and coworkers reported the first photoelectron spectroscopy experiments on the F^–^(H_2_O) at 6.42 eV,^34^ and the resulting fragmentation processes were studied by direct molecular-dynamic simulations.^35^ Nesbitt and coworkers carried out an extensive series of crossed-beam experiments on the F + H_2_O/D_2_O reaction using a combination of laser-induced fluorescence^26,27^ and high-resolution IR-laser absorption techniques for product detection.^28^ Despite the collision energies (∼0.25 eV) of these experiments falling well below the adiabatic barrier to the higher-lying A state, both OH(^2^Π_3/2_) and OH(^2^Π_1/2_) products were observed with a branching ratio of 0.69 : 0.31, suggesting that nonadiabatic surface-hopping occurs in the exit channel.^27^ The HF products were found to exhibit a vibrational population inversion, with the OH product behaving essentially as a spectator to the reaction.

The structure and energetics of the F^–^(H_2_O) anion itself have garnered significant attention from both experimental and theoretical perspectives.^36–49^ Johnson and coworkers used vibrational predissociation of F^–^(H_2_O)·Ar complexes to measure infrared spectra over 600–3800 cm^–1^, producing spectra dominated by the ionic hydrogen bond (IHB) that shuttles the shared proton between the F and O atoms.^38,41,42,44^ Features attributed to the fundamental and overtone of this mode were found to be strongly blue-shifted upon complexation with Ar, while Cl^–^(H_2_O)·Ar and Br^–^(H_2_O)·Ar are slightly red-shifted for the comparable mode.^42^ The strong blue shift was attributed to charge localization on F^–^ in the ground state, giving the anion F^–^···H–OH character. Excitation of this ionic OH stretch leads to vibrationally induced intracluster proton transfer, forming an FH···OH^–^ structure and resulting in a large anharmonicity.^44^ The competing effects of these different charge-localization states were proposed to result in the increased complexity of the F^–^(H_2_O)·Ar spectrum, with the large blue shift contributing to the significant experimental uncertainty in the frequency of the overtone of the IHB in the binary anion complex, F^–^(H_2_O)(2*ν*_IHB_).^42^

These phenomena have made theoretical treatments of this system challenging and highlighted the need for accurate PESs and a full treatment of coupled motions. One dimensional analysis of the intramolecular proton transfer coordinate in F^–^(H_2_O) shows this delocalized charge state as a shelf in the PES contributing strongly to large anharmonicities for this IHB mode. Although calculated vibrational frequencies using this reduced-dimensionality approach were found to yield reasonable results, it was determined that a 2D model based on coupling between the OF stretch and the motion of the shared proton gave the most accurate results compared to the available experimental data for the F^–^(H_2_O) system.^44^ A number of recent theoretical studies have examined the vibrational structure of F^–^(H_2_O) at a much higher level of theory,^45,47,49^ including recent full dimensionality simulations of the photodetachment spectra of vibrationally excited F^–^(H_2_O), showing that excitation of the IHB accesses the more charge delocalized FH···OH^–^ structure of the anion resulting in a change in the photoelectron spectrum.^48^ The effects of excitation of this anion IHB mode on the dissociative photodetachment of F^–^(H_2_O) is the focus of the present study.

In the following, we present a combined experimental – theoretical study of the effect of vibrational excitation of precursor anions on neutral-dissociation dynamics using PPC spectroscopy and 6D quantum dynamics calculations on *ab initio* anionic and neutral PESs. As outlined above, the neutral reaction coordinate is essentially along the motion of IHB in the parent anion. Thus, excitation of 2*ν*_IHB_ in F^–^(H_2_O) is expected to have a significant impact on the reactivity and product energy disposal. The experimentally observed effects of precursor anionic vibrational excitation on the dissociation dynamics of neutral F·H_2_O are presented and discussed in light of the extensive theoretical predictions.

## 2. Experimental methods

**Fig. 2:**
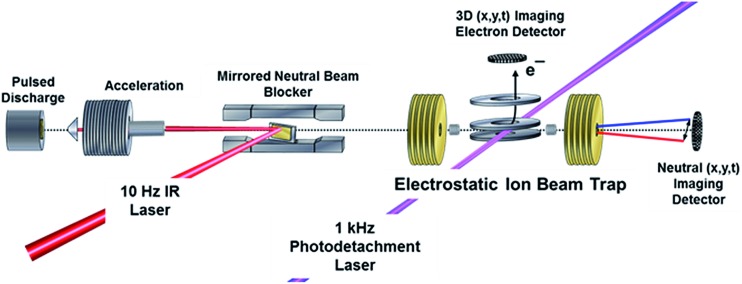
Schematic overview of the PPC spectrometer with mirrored neutral beam blocker. Ions are produced using a pulsed-valve with plasma discharge, accelerated to 7 keV and re-referenced to ground. The fast ion-beam is then irradiated with a laser pulse using a gold mirror placed along the beam axis. The ions are “bumped” over this mirror before proceeding to the EIBT where the PPC experiments are then carried out.

Experiments were carried out on a PPC spectrometer, shown schematically in Fig. 2, and described in detail elsewhere.^14,50,51^ Precursor F^–^(H_2_O) anions at *m*/*z* = 37 were synthesized in a coaxial-pulsed plasma discharge, stabilized/activated by a 1 keV electron beam acting on a pulsed supersonic expansion of C_2_F_6_/HeNe (20%/80%) passed over a 10% NH_4_OH/H_2_O mixture operating at 20 Hz. The resulting ions were skimmed, accelerated to 7 keV, re-referenced to ground in a 30 cm long tube, and mass-selected by time-of-flight (TOF). The ion packet was then injected into a cryogenically cooled electrostatic ion-beam trap (EIBT), where it was phase-locked to the output from a Ti:sapphire regenerative amplifier (Clark MXR CPA-2000; *λ* = 775.5 nm, repetition rate 1037 Hz, pulse width 1.1 ps) using an RF oscillator. The ion packet was then repetitively probed perpendicularly using third-harmonic radiation (258.5 nm, *hν*_UV_ = 4.80 eV) over a 48 ms trapping period. The resulting photoelectrons were collected on an event-by-event basis, extracted orthogonal to the ion- and laser-beam axis, and imaged onto a time- and position-sensitive detector.

Information on the center-of-mass (CM) electron kinetic energy (eKE) and laboratory-frame recoil angle were obtained from the arrival time and position for each photoelectron. When appropriate, optimal resolution was achieved by equatorially slicing the resulting photoelectron spectrum, ensuring selection of only those photoelectrons with minimal *z*-velocity. This imposed detector acceptance function (DAF) necessarily results in a reduction of signal intensity at larger photoelectron velocities, which can be corrected for by dividing the experimental intensity distribution, *N*(e_KE_), by the acceptance function of the *z*-velocity slice to provide DAF-corrected intensity distributions, *P*(eKE).^52^ Calibration of the photoelectron detector using F^–^ resulted in a sliced *z*-velocity component resolution of Δ*E*/*E* ∼ 3.8% (unsliced Δ*E*/*E* ∼ 7.2%) at 1.4 eV electron kinetic energy.

Neutral photofragments recoil out of the EIBT in a cone along the propagation direction of the ion beam, and were detected in coincidence with the photoelectron using a four-quadrant time- and position-sensitive multiparticle detector. This allows calculation of the product masses and kinetic energy release (KER) for each event containing momentum-matched neutral particles. Calibration of the multiparticle neutral detector using the dissociative photodetachment of O_4_^–^ yielded a kinetic energy release resolution of Δ*E*/*E* ∼ 10%.^50^ The mass resolution *m*/Δ*m* ∼ 15 does not allow for a direct discrimination of F + H_2_O *versus* HF + OH products.

Observation of the effect of vibrational excitation of precursor ions was achieved by irradiating the ion packet with infrared (IR) light coaxially in a counter-propagating fashion prior to injection into the EIBT as described in ref. 14 and shown in Fig. 2. Due to limitations in the IR-laser power at the *ν*_IHB_ fundamental of the anion, these experiments were carried out at 2*ν*_IHB_. A precise experimental number for this transition has not been measured, with reported values in the range 2815–2930 cm^–1^.^38,42,44^ Earlier theoretical values, which are highly sensitive to level of theory and anharmonic treatment, covered an even larger range: 2844–3055 cm^–1^.^36,39,44,45,47,48^ The data presented here were taken at *hν*_IR_ = 2885 cm^–1^, extrapolated back to the uncomplexed F^–^(H_2_O) from the vibrational-predissociation spectra of F^–^(H_2_O)·Ar_*n*_ clusters.^38,42^ The extrapolated value of 2885 cm^–1^ for 2*ν*_IHB_ in the anion is slightly lower than the reported value of 2905(20) cm^–1^ for neat F^–^(H_2_O) given by Johnson and coworkers,^42^ but within the stated experimental uncertainty. Thus, although the clearest effects were observed at 2885 cm^–1^, additional data was collected both at 2900 cm^–1^ and at 2872 cm^–1^, as presented in the (ESI†). The anion packet was irradiated at 2885 cm^–1^, 2872 cm^–1^, or 2900 cm^–1^ using the tunable output of a 10 Hz Nd:YAG (Surelite III EX) pumped KTP/KTA optical-parametric oscillator/optical-parametric amplifier (OPO/OPA) system (Laser Vision, 100–300 mW, 5 ns FWHM, bandwidth ∼3 cm^–1^). The IR wavelength *λ* was set using the OPO signal and idler wavelength, measured using a spectrometer (Ocean Optics HR2000+) and independently calibrated using photoacoustic spectroscopy on CH_4_. Counter-propagation of the IR pulse relative to the fast-ion packet gives rise to a Doppler shift of 2 cm^–1^, corrected for prior to data collection. Source fluctuations were mitigated by data collection in an interleaved fashion, where ion source and PPC measurements were run at a 20 Hz duty cycle, all the while synchronized with the 10 Hz IR laser system such that every other 48 ms trapping cycle was carried out on an IR-irradiated anion packet. IR-irradiation of the anions occurred ∼10 μs before the first photodetachment laser shot in the EIBT. These interleaved IR on/off data sets were separated during post-processing, calibrated, and analyzed in the usual way. Effects of IR excitation are analyzed by examining difference spectra (IR–no-IR), and the experimental uncertainty for each is determined on a bin-by-bin basis, using 
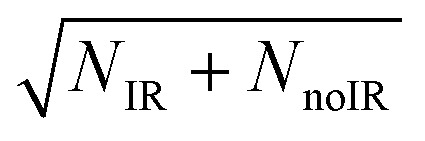
, where *N*_IR_ and *N*_noIR_ correspond to the number of events in the IR and no-IR spectra. Based on analysis of the stable channel discussed in the ESI,† a best estimate for the excitation fraction of the anion packet is ∼4%. Although the best photoelectron resolution is typically achieved by slicing along the *z*-velocity component, the subsequent DAF correction resulted in increased noise at larger eKE when determining the difference spectra. Consequently, all difference spectra presented here use the full unsliced data set.

## 3. Theory

The theoretical results reported here were obtained from full-dimensional quantum dynamical calculations using the *ab initio* based global PESs of the lowest two electronic states of FH_2_O,^30,31^ and a semi-global PES for the anion F^–^(H_2_O).^13^ Both the X and A states of the neutral are responsible for the highly exothermic F + H_2_O ↔ HF + OH reaction, but the latter has a much higher barrier. Both PESs are dominated by a deep post-transition state well, featuring a hydrogen-bonded FH···OH complex. The equilibrium geometry of the anion is close to the transition-state geometry of the neutral PESs, providing an ideal system for transition-state spectroscopy.^13^ The quantum dynamical Hamiltonian is written in either the diatom–diatom Jacobi or atom–triatom Radau–Jacobi coordinates (Fig. S1 in ESI†) and the total angular momentum *J* is set to zero.^23^ The *J* = 0 assumption is reasonable as the experimental temperature of the anion is expected to be < 100 K based on measurements of the photodetachment of OH^–^ under similar conditions,^53^ but there is no question that a range of rotational levels in the F^–^(H_2_O) anion will be populated. The photodetachment is modeled within the Condon approximation, in which the ejection of the electron from the anion is approximated by a vertical excitation to the neutral species, and the electron carries away no angular momentum in s-wave photodetachment. Quantum dynamics on the neutral PESs were followed using the Chebyshev propagator,^54^ and final state distributions were obtained by projecting the wavepacket onto the product states.^51,55^

**Fig. 3:**
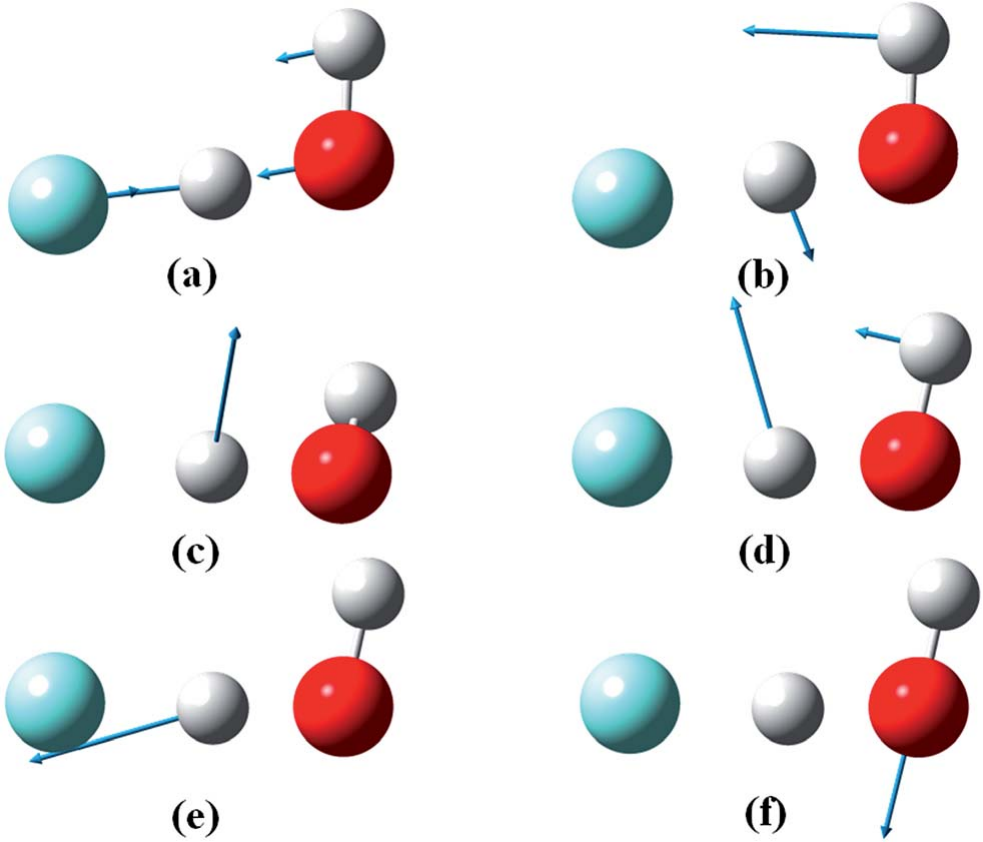
Normal modes of the F^–^(H_2_O) anion. (a) Ion–water stretch, *v*_IW_, (b) in-plane wag, *v*_IP_, (c) out-of-plane wag, *v*_OOP_, (d) water bending, *v*_B_, (e) ionic hydrogen bond, *v*_IHB_ and (f) free OH stretch, *v*_F_.

In the process of carrying out the supporting theoretical results reported here, exact calculations of the low-lying vibrational energy levels were determined on the F^–^(H_2_O) anion PES. The results are listed in Table 1 with the corresponding normal mode vectors displayed in Fig. 3. The agreement with experimental^44,45^ and previous theoretical results^47,49^ is quite good. The vibrational state excited in the experiment is identified as the overtone of the ionic hydrogen bond at 2*ν*_IHB_ = 2838 cm^–1^. This is 47 cm^–1^ below the estimated experimental value of 2885 cm^–1^, but given the 50 cm^–1^ splitting observed in the IR absorption spectrum in the region of 2*ν*_IHB_,^42^ this is reasonable agreement. Table 1 also shows that the fundamental frequencies for the out-of-plane wagging and water bending modes are such that a combination of these excitations is nearly degenerate with the first overtone of the ionic hydrogen bond, contributing to the complexity of the spectrum in this energy range.

**Table 1:** Comparison of calculated vibrational energy levels of three low-lying vibrational levels with experimental band origins for the F^–^(H_2_O) anion (see caption for Fig. 3)

Mode	Expt.^45^	Expt.^44^	Theory (VCI on KTCB PES)^47^	Theory (SLBCL PES)^49^	Theory (KTCB PES, this work)	Theory (new PES, this work)
*ν*_IW_ (F^–^–HOH)	426.65	—	433.24	423.5	432.67	426.87
*ν*_IP_	576.27	—	566.61	560.7	565.96	563.18
2*ν*_IW_	829.10	—	836.86	823.2	835.68	828.74
*ν*_OOP_	1184.36	1083–1250	1146.62	1156.1	1154.85	1173.74
*ν*_IHB_	1464.54	1430–1570	1456.71	1477.9	1464.01	1468.04
*ν*_B_	1653.56	1650	1623.25	1716.8	1642.74	1663.28
2*ν*_IHB_	2915.91	2815–2930	2872.49	2837.3	2847.03	2838.01

The numerical parameters used in the dynamical calculations are collected in Table S1.† Due to the strong long-range interaction between HF and OH species, a large diatom–diatom distance must be used in the calculations. To reduce the size of the basis set, an L-shape grid and the vibrational basis were adopted. Due to exceedingly long lifetimes of the Feshbach resonances in the post-transition state well, the propagation was terminated at 20 000 to 25 000 steps, which roughly correspond to 1 ps. The energy spectrum of the final wave packet was analyzed by taking the Fourier transform of the autocorrelation function.^54^

## 4. Results

The most unbiased approach to examining the effects of vibrational excitation on the dynamics of the reaction in the present case of limited excitation of the parent anion beam is to analyze difference spectra from the interleaved IR laser-on/IR laser-off measurements. In the limit of no correlations between the subset of ions excited, their decay rates in the EIBT, the dissociation rate of the neutral complexes, and the subsequent dissociation dynamics, these difference spectra should correspond to the difference of the theoretical IR and no-IR spectra. If the F^–^(H_2_O) spectrum is *a*, and the F^–^(H_2_O)(2*ν*_IHB_) spectrum is *b*, the difference spectrum for an excitation fraction of *f* will be given by the difference *D* = (((1 – *f*)*a* + *fb*) – *a*) = *f*(*b* – *a*) ∝ *b* – *a* within a normalization constant. The quantum dynamics calculations show that these results are compared to cover ∼1 ps wavepacket propagation, and are thus most sensitive to prompt dissociation processes, so the present work focuses on a comparison of theory with the two-dimensional PPC difference spectrum as well as the difference spectrum for the total kinetic energy release, *E*_TOT_, where *E*_TOT,i_ = eKE_i_ + KER_i_ is determined for each event i and summed. Difference photoelectron spectra for stable and dissociative neutral products are provided in the ESI.† As noted in the experimental section, resolution has been sacrificed in favor of statistics in the work presented here, and no slicing of the photoelectron velocity distributions are used, so the resolution of the PPC and *E*_TOT_ spectra are reduced relative to the study of F^–^(H_2_O) without IR excitation in ref. 13.

**Fig. 4:**
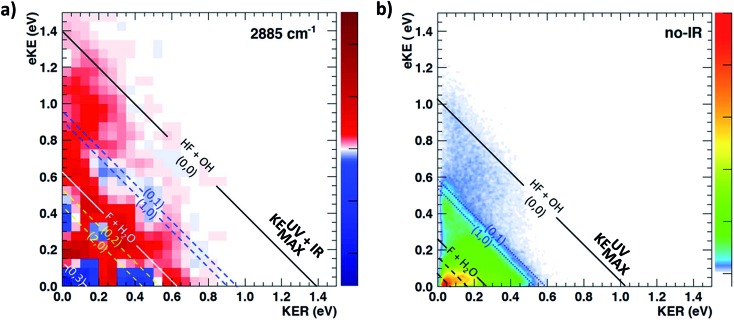
Photoelectron photofragment coincidence (PPC) difference spectrum for (IR–no-IR) data for precursor anions excited with *IR* = 2885 cm^–1^ (a). The PPC spectrum for the no-IR F^–^(H_2_O) data alone is shown in the right frame (b). For the no-IR spectrum, black solid diagonal lines indicate the energetic limits, KEUVMAX, for dissociation into HF + OH + e^–^ and F + H_2_O + e^–^ fragments, respectively; dashed lines indicate vibrationally excited product states. For the difference spectrum (a), these energetic limits have been shifted higher by the IR photon energy relative to the no-IR PPC spectrum; blue areas indicate suppression and red areas indicate enhancement relative to the no-IR spectrum.

The difference PPC spectrum illustrating the effect on the dissociation dynamics of the nascent FH_2_O neutral complex following photodetachment of F–H–O overtone-excited anions at *hν*_IR_ = 2885 cm^–1^ is shown in Fig. 4(a). For reference, the PPC spectrum of F^–^(H_2_O) without IR excitation taken simultaneously with this data is shown in Fig. 4(b). In the ESI, Fig. S6† shows PPC difference spectra for IR photon energies of 2885, 2872 and 2900 cm^–1^, as well as a null difference spectrum, to provide a measure of the magnitude of the effect. The 2885 cm^–1^ spectrum exhibits the strongest effect of vibrational excitation, although the 2872 cm^–1^ spectrum also shows statistically significant enhancement above a total energy of 1.0 eV. The 2885 cm^–1^ spectrum exhibits the strongest effect and is therefore the focus of this work. All diagonal energetic limits for the dissociative pathways for both the no-IR and IR cases are tabulated in Table S2,† with the limits in Fig. 4(a) shifted above those in Fig. 4(b) by the addition of *hν*_IR_ such that *hν* = *hν*_uv_ (4.80 eV) + *hν*_IR_ (0.36 eV) = 5.16 eV is the total available photon energy. In both frames, the rightmost diagonal line corresponds to the energetic limit for the HF + OH products formed in their ground rotational and vibrational states, denoted KEUV+IRMAX and KEUVMAX for the IR-excited and no-IR cases, respectively. For convenience, product states are referenced herein in the general form (*n*_HF_, *n*_OH_). The energetics for formation of HF(*n*_HF_ = 0) + OH(*n*_OH_ = 1) and HF(*n*_HF_ = 1) + OH(*n*_OH_ = 0) products, denoted (0, 1) and (1, 0) respectively, are denoted in the difference spectrum by dotted blue lines. The black diagonal line at 0.26 eV in the no-IR PPC spectrum, and black diagonal at 0.62 eV in the difference spectrum correspond to formation of the reactants F + H_2_O. Both spectra reveal a strong preference toward vibrational excitation in the products, consistent with the HF product fragment being formed with considerable internal excitation, in agreement with previous experimental studies.^13,28^

The difference spectrum in Fig. 4(a) shows clear effects from IR excitation of 2*ν*_IHB_ in F^–^(H_2_O). The most obvious difference is the positive red band of events above the no-IR KEUVMAX (1.03 eV, not shown on the difference spectrum for clarity, but shown in Fig. 4(b)) and below the IR-excited product KEUV+IRMAX line (solid black diagonal line). This new band in the IR excitation difference spectrum is consistent with accessing the ground vibrational state of the HF + OH dissociation pathway, where the additional energy from the IR photon has been partitioned into translational motion of the resulting photoelectron, appearing at greater eKE but identical KER as the no-IR case. This is essentially a classic ‘hot-band’ in photoelectron spectroscopy, where vibrational energy in the anion is converted to eKE, leaving the dissociating molecular fragments with less total energy. This band consists of two broad features, one closer to the KEUV+IRMAX limit at higher eKE and smaller KER, and a second, weaker feature appearing at lower eKE, just above the energetic limit for HF(*n*_HF_ = 0) + OH(*n*_OH_ = 1) (blue dashed line), with a long tail extending out to KER ∼0.65 eV. The first feature trails off at KER ∼0.40 eV, as in the no-IR PPC spectrum. The second feature, with the long KER tail, could be interpreted as rotational excitation in at least one of the HF and/or OH products. The large KER may result from increased Franck–Condon overlap with the more-repulsive A-state barrier that plays an important role in the (0, 1) and (1, 0) channels for the reaction with no vibrational excitation,^13^ but the low intensity means that these features are not as statistically significant.

The next prominent diagonal band of enhanced (red) signal in Fig. 4(a) appears below the energetic limit for (1, 0) excitation, lying just above the adjusted UV + IR energetic limit for the F + H_2_O reactant channel. As in the no-IR spectrum, this band covers the full range of allowed eKE and KER, notably without the distinct KER cutoff seen in the (0, 0) features. The peak enhancement signal falls below the energetic limits for the (1, 0) or (0, 1) product states by 0.2–0.3 eV, which can also be rationalized with production of these vibrationally excited products with significant rotational excitation.

The limited product mass resolution under the current experimental conditions prevents resolution of the F + H_2_O reactant channel from the HF + OH product channel. The energetic limit for formation of the F + H_2_O reactant-channel products is denoted by the solid white diagonal line at 0.62 eV in Fig. 4(a). The energetic limits for production of the (0, 2) and (2, 0) final states are denoted by black dashed diagonal lines. This convoluted portion of the difference spectrum is dominated by “depletion” (blue) signal. The depletion observed here corresponds to the most intense portions of the no-IR PPC spectrum, where the feature near eKE = 0.4 eV is attributed to the no-IR (1, 0) band and the long horizontal band covering the range KER = 0.0–0.4 eV has previously been observed in the no-IR spectrum as eKE = 0 eV is approached.^13^ IR excitation shifting some of these high intensity features seen in the no-IR spectrum makes it is plausible that depletion signal would be observed in these regions in the PPC difference spectrum. However, these features are also less significant statistically as will be further discussed in the next paragraph.

**Fig. 5:**
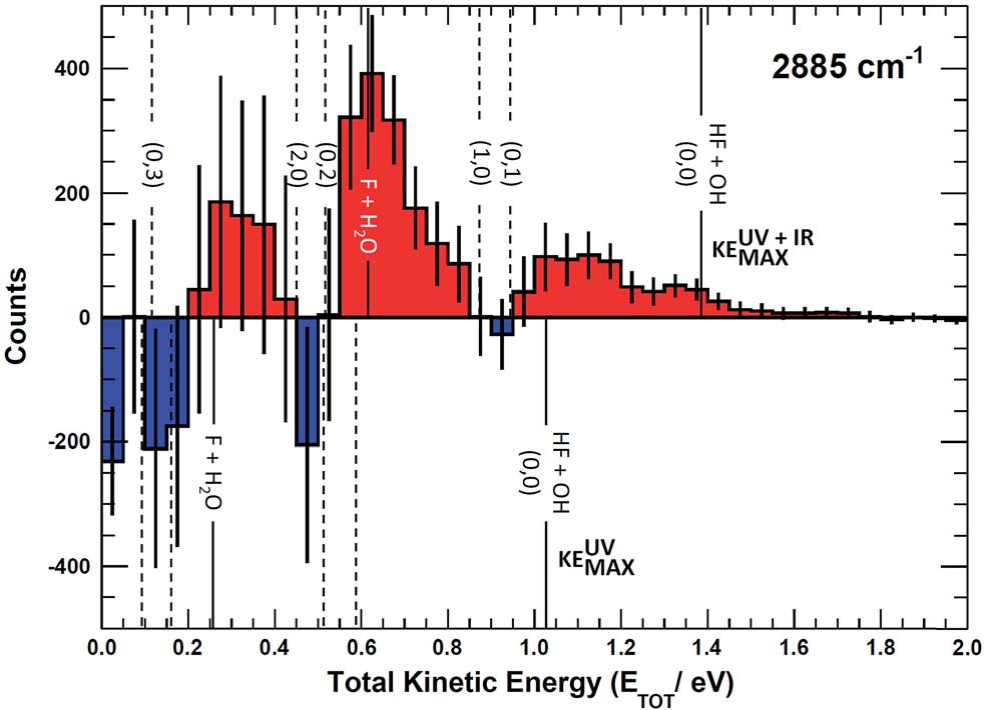
Total kinetic energy (*E*_TOT_ = eKE + KER) difference (IR–no-IR) spectra for *IR* = 2885 cm^–1^, showing IR-excited (top) and no-IR (bottom) energetic limits. Blue and red areas indicate suppression and enhancement, respectively, relative to the no-IR spectrum. Error bars correspond to 
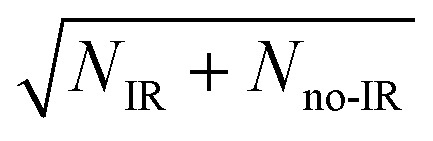
 for each bin.

Integrating along the diagonal produces the *E*_TOT_ difference spectrum, shown in Fig. 5, with the integrated IR and no-IR spectra providing a direct measure of the product state distributions prior to taking the difference. The energetic limits for dissociation to the respective pathways are the same as for the PPC spectra, and are indicated here with vertical lines. The top panel shows signal enhancement in red, with the vertical lines corresponding to the IR-excited energetics. The bottom panel shows signal depletion in the difference spectrum with the no-IR KE_MAX_ limits indicated. Error bars represent the statistical uncertainty and are calculated for each bin using
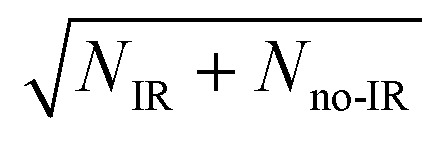
. Again, the red enhancement data above the no-IR KEUVMAX at *E*_TOT_ = 1.03 eV provides unambiguous evidence for 2*ν*_IHB_ excitation of the precursor anions in a broad band, consistent with the possible rotational excitation in one or both diatomic products. This is also true for the enhancement data in the (1, 0) channel, spanning the (1, 0) to (2, 0) energy range and peaking at the energetic limit for appearance of the F + H_2_O reactant channel. This is consistent with vibrational excitation of the anion leading to enhanced production of the reactant channel following photodetachment. The trend of apparent rotational excitation in the products does not appear in the case of the (2, 0) channel, which peaks closer to the (2, 0) limit. At the threshold for production of OH(*n*_OH_ = 1), the (0, 1) limit, there is a blue suppression feature, indicating that 2*ν*_IHB_ excitation does not favor production of vibrational excitation in the ‘external’ OH bond that becomes the nascent OH product after dissociation. Obviously, as eKE decreases, the product density of states increases and assignment of these overlapping spectral features becomes less conclusive. A second point is that the error bars below eKE = 0.5 eV become comparable to the observed differences, so these are not statistically significant.

## 5. Discussion

**Fig. 6:**
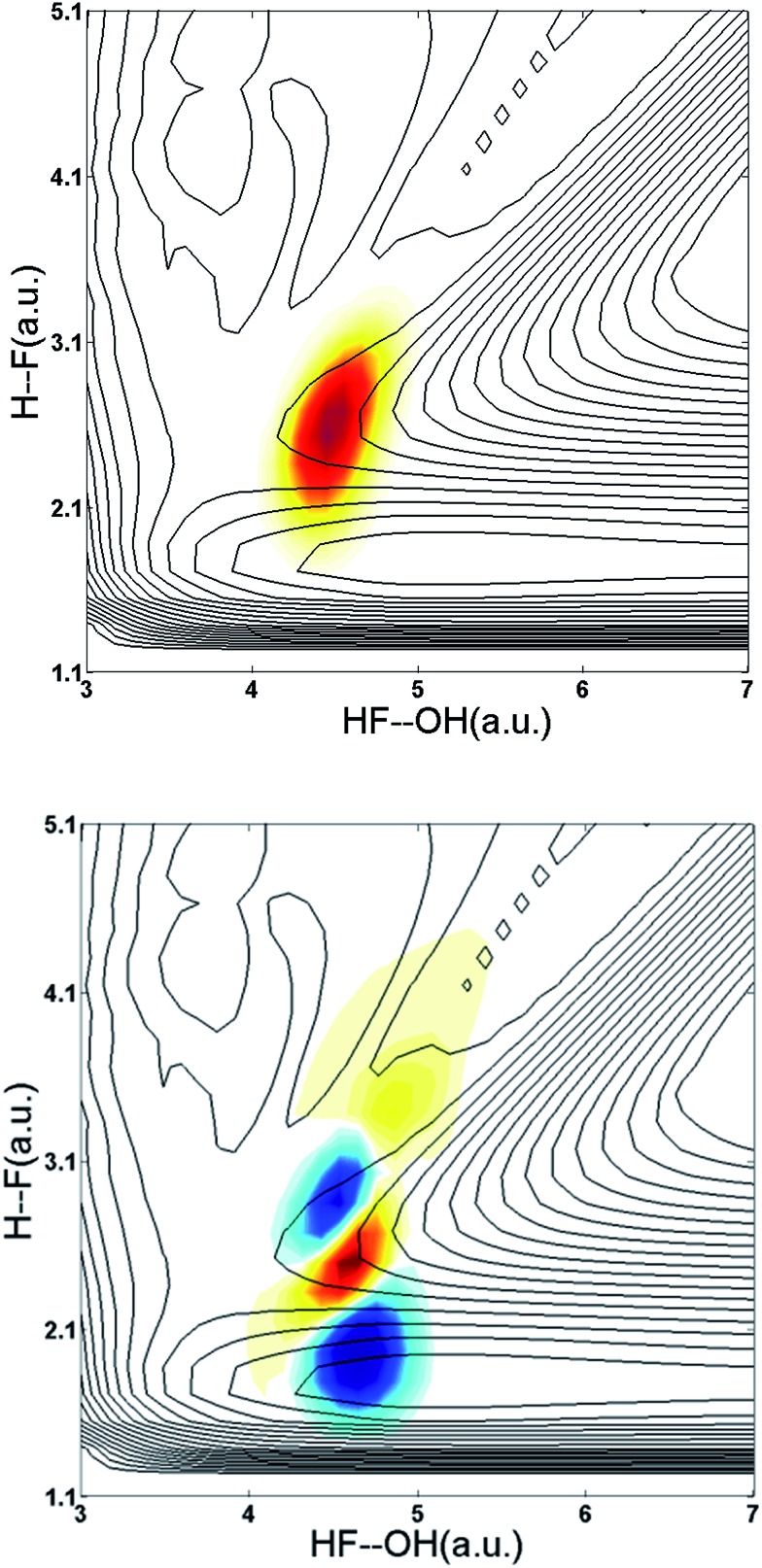
Wavefunctions of the ground state and the overtone 2*ν*_IHB_ of the F–H–O ionic hydrogen bond of the F^–^(H_2_O) anion superimposed on the X state PES.

This system is an ideal one for studying the transition-state dynamics and the effect of vibrational excitation of the shared hydrogen atom on the neutral F + H_2_O → HF + OH reaction. This can be most clearly seen by examining the wavefunctions of ground state F^–^(H_2_O) and F^–^(H_2_O)(2*ν*_IHB_) anions superimposed on the X state PES, as shown in Fig. 6. The two anion wavefunctions access significantly different coordinate spaces on the neutral PESs, providing an expectation that a significant vibration-induced effect on the reaction dynamics will be observed. In the anion ground state, the negative charge is located primarily on the F atom, giving the anion an F^–^···H···OH type structure. Photodetachment from this ground state probes near the transition-state geometry on the neutral PES. With additional internal energy in the IHB mode, the anion assumes a more charge-delocalized structure, FH···OH^–^.^44,48^ This increased FH···OH^–^ character might be expected to provide better Franck–Condon overlap with the product-channel FH–OH complex well and the product-channel asymptote, but the quantitative effects are more complex. The additional available energy in the system is significantly above the X state barrier, reaching near the top of the A state barrier as shown in Fig. 1. As in the case of the earlier study of this system without vibrational excitation,^13^ the dynamical calculations revealed that the dissociation of the neutral FH_2_O complex prepared by photodetaching F^–^(H_2_O) produces both F + H_2_O and HF + OH products, with significant internal excitation. The branching ratio between reactants and products as well as the vibrationally resolved branching percentages (not energy resolved) for HF + OH are summarized in Table 2. These results show that dissociation to F + H_2_O reactants more than triples to 37% for photodetachment to the X state while the A state contribution increases from 0 to 14% upon excitation of 2*ν*_IHB_.

**Table 2:** Calculated fractions of vibrational state-resolved channels in the photodetachment of ground state F^–^(H_2_O) anion and F^–^(H_2_O) with 2*v*_IHB_ overtone excitation. The contributions of the X and A states are reported separately

Channel	Fraction (%)		(*n*_HF_, *n*_OH_)	Fraction (%)	
	Ground state F^–^(H_2_O)	2*v*_IHB_		Ground state F^–^(H_2_O)	2*v*_IHB_
X: F + H_2_O	13%	37%			
X: HF + OH	87%	63%	(0, 0)	9.4%	19.3%
			(0, 1)	2.7%	0.8%
			(0, 2)	1.6%	0.3%
			(0, 3)	0.0%	0.1%
			(1, 0)	60.3%	13.4%
			(1, 1)	2.6%	2.7%
			(2, 0)	9.6%	26.3%
A: F + H_2_O	0%	14%			
A: HF + OH	100%	86%	(0, 0)	24.7%	60.7%
			(0, 1)	3.2%	1.2%
			(0, 2)	0.0%	0.1%
			(0, 3)	0.0%	0.0%
			(1, 0)	71.8%	18.0%
			(1, 1)	0.0%	0.6%
			(2, 0)	0.2%	5.0%

**Fig. 7:**
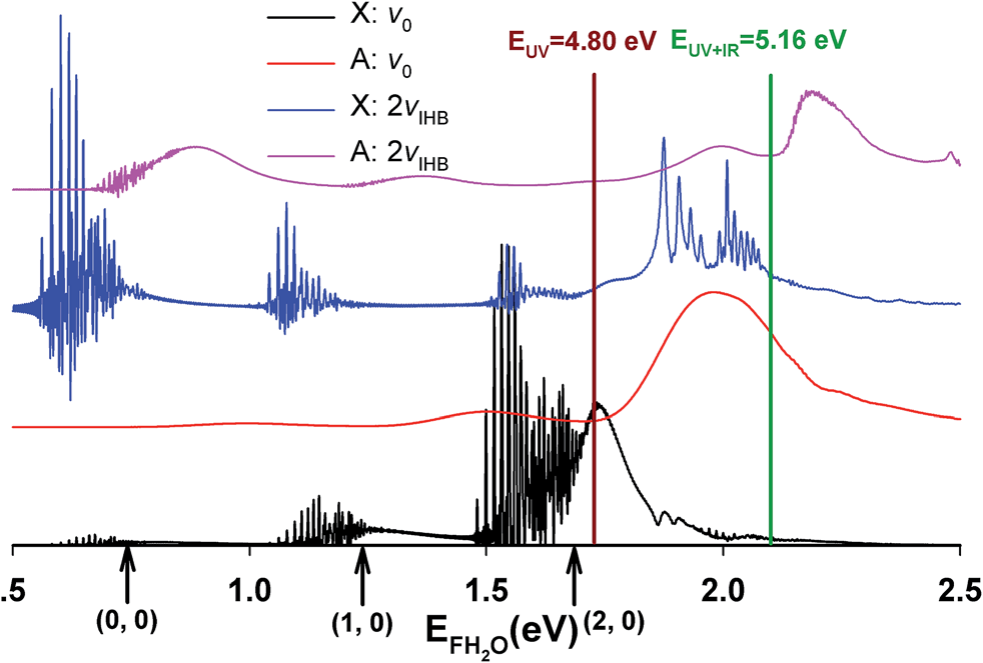
Calculated photoelectron spectra for F^–^(H_2_O)(2*ν*_IHB_). For comparison, the corresponding spectra for the ground vibrational state of the anion are also included.

The calculated X and A state photoelectron spectra for ground state F^–^(H_2_O) and F^–^(H_2_O)(2*v*_IHB_) are shown in Fig. 7. The energy zero is defined as the minimum of the FHOH product-channel H-bonded complex of neutral FH_2_O. The overall shapes of the spectra are similar, but the intensities vary considerably, reflecting the access of different regions of the neutral PESs. For the X state, the distinct bands separated by about 0.5 eV can be clearly assigned to different product HF vibrational levels. The fine structure within each band is due to Feshbach resonances, as pointed out in our earlier work.^14,23^ The A state spectra are much smoother, indicating fast and more direct dissociation, due to the more repulsive PES produced by the higher barrier in the A state shown schematically in Fig. 1, with the corresponding bands generally higher in energy than those on the X state spectrum. In the same figure, the photon energies of the two experiments (UV and UV + IR) are indicated by vertical lines. It is worth noting in passing that these calculated spectra are very different from the recent report using the reflection principle,^48^ which does not take into consideration the multidimensional neutral dynamics following the initial photodetachment.

**Fig. 8:**
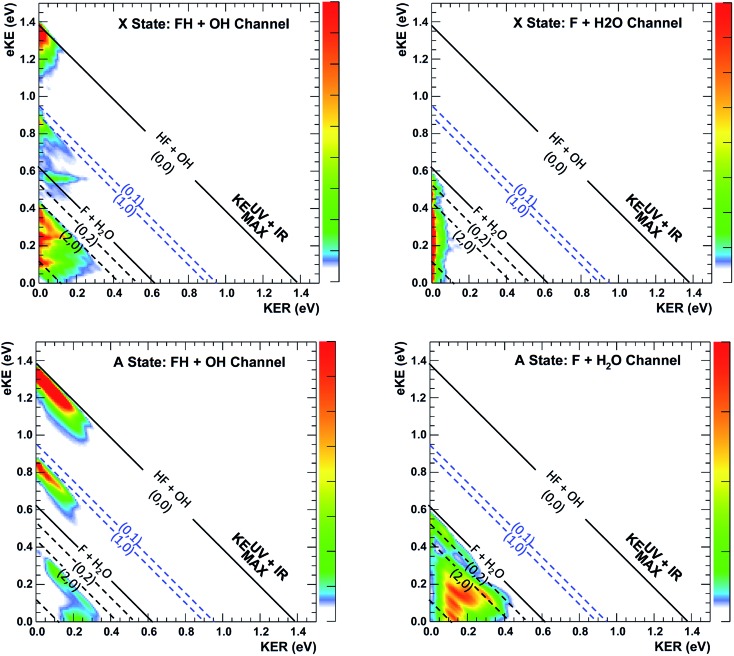
Channel-resolved PPC spectra based on quantum dynamical calculations for the dissociation of FH_2_O prepared by photodetachment of F^–^(H_2_O)(2*ν*_IHB_). Energetic limits are established by experimental data as documented in Table S2,† with the solid KE_MAX_ limits for production of HF(*v* = 0) + OH(*v* = 0) and F + H_2_O in the product ground states shown as the solid lines at 1.39 and 0.62 eV. The corresponding calculated values are 1.34 and 0.56 eV, based on calculations on the PIP potential energy surface, with the asymptotic levels calculated at 12 Bohr. The (*n*_HF_, *n*_OH_) product states KE_MAX_ limits for(0, 0), (0, 1), (1, 0), (0, 2), (1, 1), (2, 0) and (0, 3) are calculated at eKE = 1.34, 0.90, 0.85, 0.47, 0.40, 0.37, 0.07 eV, respectively, and compare well with the diagonal limits based on experimental data used for the dashed diagonal limits in the figure.

A key element of the present comparison of experiment and theory is the explicit theoretical treatment of the neutral dynamics on the ps timescale, including the role of repulsive states, Feshbach resonances, and reactant and product complexes. The calculated PPC spectrum for F^–^(H_2_O)(2*v*_IHB_) shown in Fig. 8 captures the breadth of these results, resolved into contributions from the X and A state into the reactant F + H_2_O and product HF + OH channels. Integration over these spectra provides the product state distribution results in Table 2, showing that the HF + OH channel dominates, particularly in the A state, but more F + H_2_O flux is seen for the vibrationally excited anion. In the HF + OH channel, the HF product is highly excited while the OH product in mostly in its ground vibrational state. The larger population in the HF(*n*_HF_ = 0) product channel stems from an improvement in the Franck–Condon overlap, but the enhancement of the HF(*n*_HF_ = 2) product channel is in part due to the higher photon energy that accesses more final states in that manifold. The large excitation in the HF vibration is due to the fact that the HF vibrational mode is well aligned with the anion vibrational excitation.^56^ As shown in Fig. 6, the initial wavepacket for F^–^(H_2_O)(2*v*_IHB_) has a large span in the H–F coordinate, leading to significant vibrational excitation in the HF product.

**Fig. 9:**
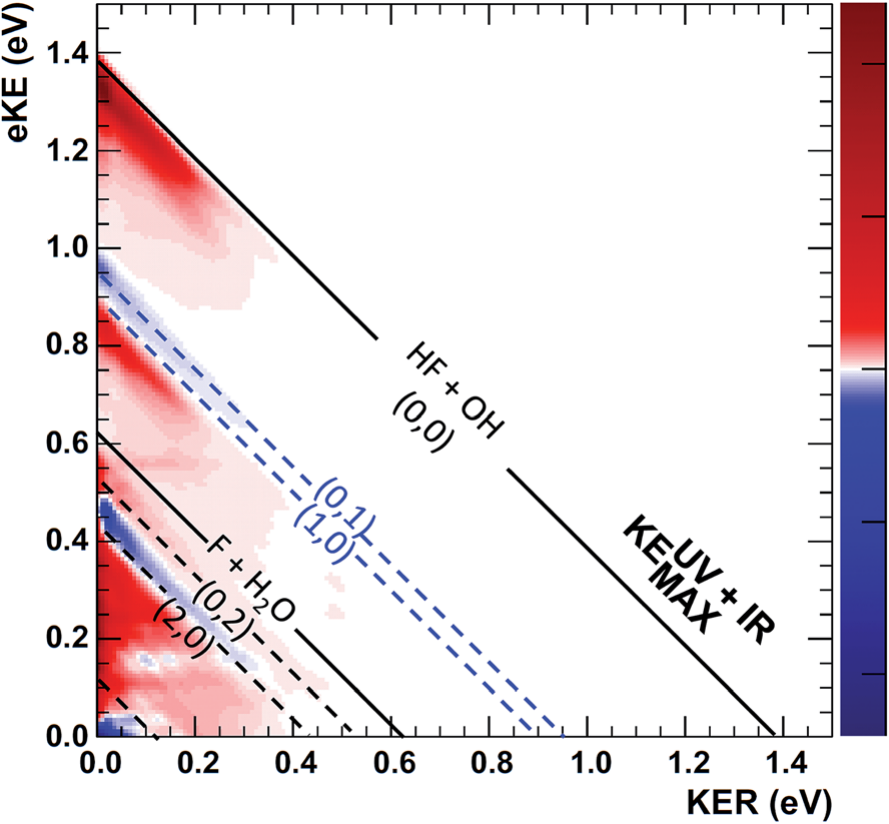
Theoretical difference (IR–no-IR) PPC spectrum, convolved with a Gaussian function with *σ* = 0.005 eV. The resolution in the experimental data is better reproduced by *σ* = 0.03 eV, but higher resolution is retained here for clarity concerning the potentially observable effects from vibrational excitation. The annotated diagonal limits show the maximum kinetic energy limits for (*n*_HF_, *n*_OH_) product states and the F + H_2_O channel.

**Fig. 10:**
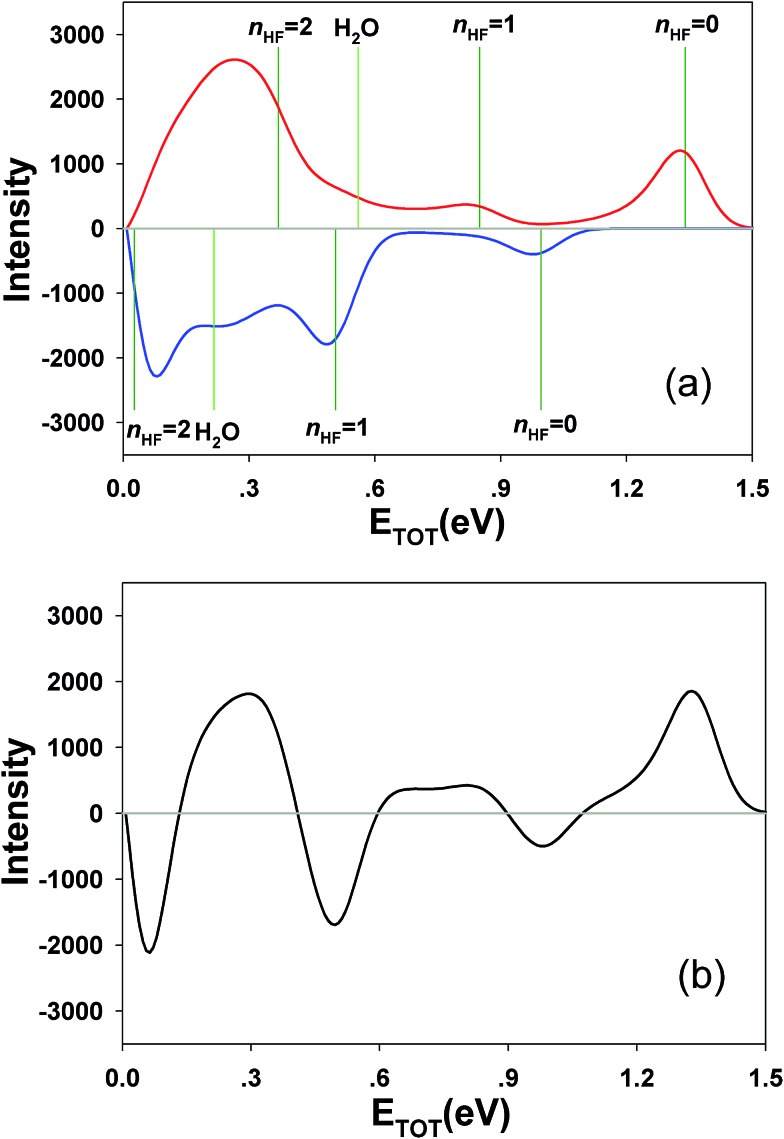
(a) Intensity distributions for vibrational ground (blue line) and excited (red line) states of anion at different total kinetic energies (*E*_TOT_ = eKE + KER), convolved with a Gaussian with *σ* = 0.03 eV. (b) Total kinetic energy (*E*_TOT_ = eKE + KER) difference (IR–no-IR) spectra for 5% IR excitation of IR signal convolved with a Gaussian with *σ* = 0.03 eV.

Given the limitations in the experiment with respect to the fraction of IHB-excited anions (∼4%) and signal-to-noise in the difference spectra, it is invaluable to have these quantum dynamics predictions of the PPC and *E*_TOT_ spectra to compare with the data. It is important to recognize two issues when comparing the experimental and theoretical PPC spectra. First, the theoretical results are obtained from a relatively short (∼1 ps) wavepacket propagation. As a result, they might differ from the experimental spectra that include events that occur over the flight time from the interaction region to the detector, 7 μs at the beam energy of 7 keV. As shown in the theoretical photoelectron spectra in Fig. S6,† dissociation on a 1 ps timescale typically involves high-energy short-lived resonances and direct processes. Indeed, an important assumption in comparing the theoretical results for the dissociation of FH_2_O and FH_2_O(2*v*_IHB_) is that the dissociation rate is equal on the 1 ps timescale. If the dissociation rate is equal and the photodetachment cross sections are identical, the experimental and theoretical results should be comparable as straightforward differences of the FH_2_O and FH_2_O(2*v*_IHB_) theoretical predictions *versus* the IR–no-IR difference spectra as discussed in the results section. Second, it is known that the anion has a complex vibrational structure,^42^ and may exhibit intramolecular vibrational energy redistribution (IVR), leading to mixing with other vibrational states nearby. In particular, as noted earlier, a combination band involving the out-of-plane wagging and water bending modes is nearly degenerate with 2*v*_IHB_, and excitation or IVR into this combination band could lead to product rotational excitation. As noted in the experimental section, the anions are excited 10 μs prior to trapping in the EIBT, and then the data is acquired over a period of 48 ms, so there is significant time for IVR to occur. In principle, this effect can be examined in the experimental data, but signal-to-noise limitations make that beyond the scope of the present work. The theoretical predictions will thus be compared directly to the experimental PPC and *E*_TOT_ difference spectra shown in Fig. 4(a) and 5 using theoretical difference spectra assuming 5% F^–^(H_2_O)(2*v*_IHB_) excitation, shown in Fig. 9 and 10, respectively.

The enhancement signal observed in the PPC difference spectrum in Fig. 4(a), appearing below the IR-excited KEUV+IRMAX and above the no-IR KEUVMAX, can come only from ground-state HF + OH products accessed after vibrational excitation of the precursor anions. This shows that vibrational energy along the proton transfer coordinate in the precursor anion can be carried away by the photoelectron in a bound-free Franck–Condon photodetachment, and is seen in both the experimental and theoretical results. This is largely because the vibrational coordinate is well aligned with the neutral reaction coordinate near the transition state. Beyond serving as proof of concept for successful preparation of F^–^(H_2_O)(2*ν*_IHB_), this band is largely consistent with the higher resolution data previously reported for the experiment with ground state F^–^(H_2_O).^13^ The signal grows in starting at the energetic limit for (0, 0) and appears as a fairly broad diagonal band. Both the X and A states contribute to the increased intensity in the (0, 0) band, with the larger KER contributions produced by dissociation on the excited A state. Dissociation on the A state yields products with a larger kinetic energy, which is consistent with the fact that the A state PES is energetically higher in the Franck–Condon region and more repulsive. The second feature appears just above the energetic limit for (0, 1) at a similar KER and has a weak and less statistically significant tail extending to larger KER. The larger KER tail is not seen in the theoretical PPC difference spectrum in Fig. 9. The fact that the second feature appears well below the product KEUV+IRMAX in the experimental spectrum is consistent with formation of these products with considerable rotational excitation in at least one of the product fragments. One important difference between theory and experiment is that the experimental PPC difference spectrum does not indicate the significant formation of rotationally cold HF + OH products in the upper-left corner of the energetically allowed region, as seen in the theoretical PPC spectra in Fig. 8 as well as the high-resolution cold F^–^(H_2_O) spectra in ref. 13. Examination of the no-IR F^–^(H_2_O) spectrum in Fig. 4(b) also shows diminished intensity in that region, suggesting that perhaps the 20 Hz ion source used in these measurements did not cool parent anion rotational states as effectively as in the earlier experiment.

Vibrational excitation in the HF product is particularly sensitive to changes in reaction dynamics, with the theoretical results indicating a suppression of HF(*n*_HF_ = 1) and enhancement of HF(*n*_HF_ = 0, 2) in both the X and the A states, as shown in Table 2. In the experimental no-IR PPC spectrum (Fig. 4(b)), HF(*n*_HF_ = 1) signal dominates, appearing as an intense spot near eKE = 0.4 eV, and as a band with a sharp onset at the threshold covering the full range of available KER as first reported in ref. 13. In the difference PPC spectrum, the enhancement signal for this channel falls noticeably below the (1, 0) limit, observed as a prominent red band that extends to lower energies beyond the F + H_2_O reactant KEUV+IRMAX= 0.62 eV (annotated solid black lines in Fig. 4(a) and 5). As such, the enhancement-signal peaking at the F + H_2_O reactant-pathway energetic limit in the difference spectra was initially attributed to increased contributions from the (1, 0) channel where products are formed with substantial rotational excitation in at least one product-channel fragment. With the reduced resolution in the present experimental results relative to ref. 13, however, some of the observed enhancement near the F + H_2_O KEUV+IRMAX limit may arise from the formation of the F + H_2_O products predicted to be significantly enhanced in the dynamical calculations for photodetachment of F^–^(H_2_O)(2*ν*_IHB_). The product distributions in Table 2 show that the (2, 0) population is enhanced by anion vibrational excitation, particularly in the X state. This appears as a large feature in the calculated PPC spectrum for the HF + OH products formed on the X state in the (2, 0) band in the upper left frame of Fig. 8. On the other hand, in the experimental data, the HF + OH signal is superimposed on the F + H_2_O signal, which is also significantly enhanced. These overlapping features make interpretation of the experimental PPC spectrum at lower eKE and KER increasingly difficult.

**Fig. 11:**
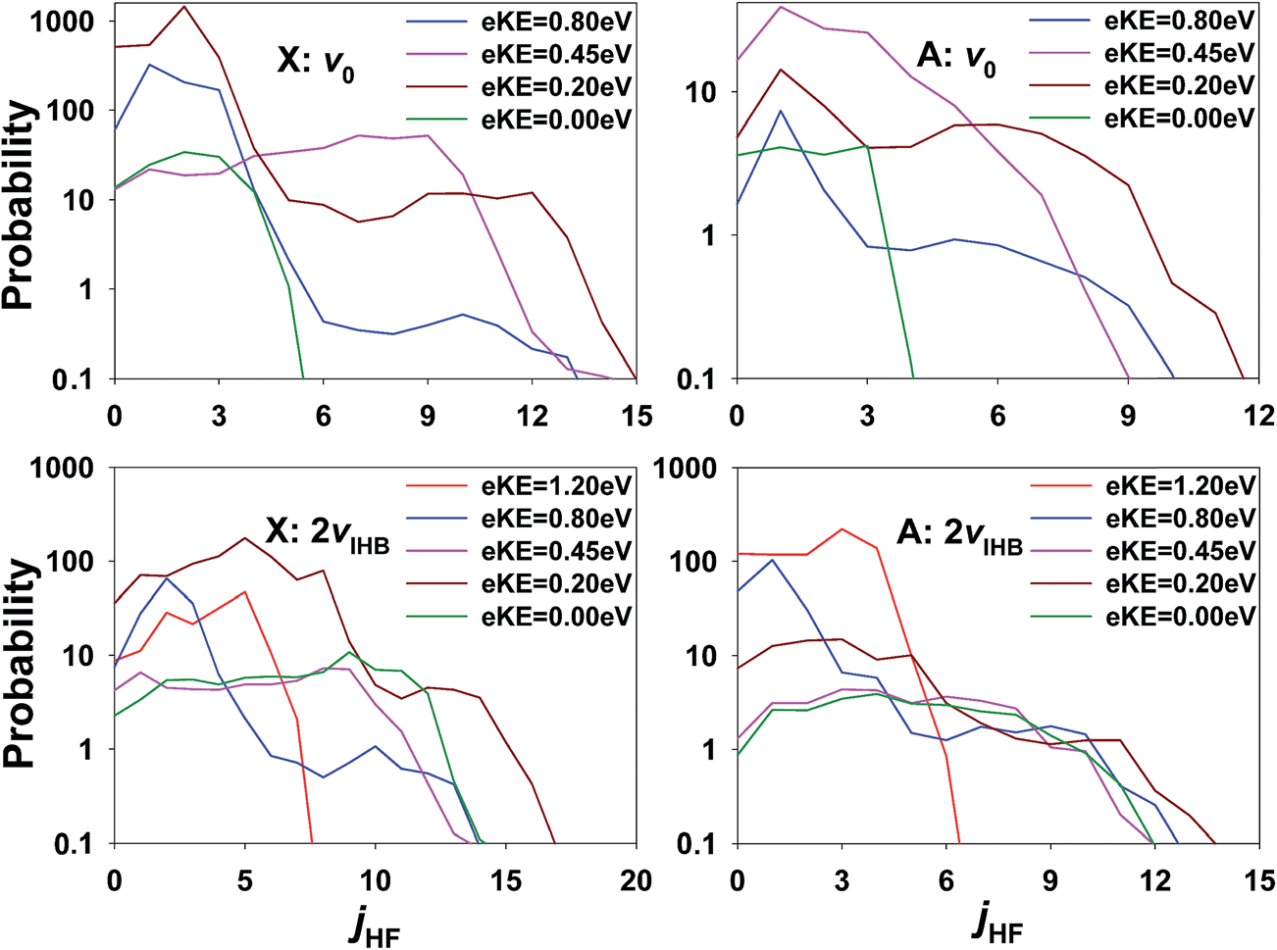
Rotational state distributions of HF and OH on the X/A state at several eKEs for ground state F^–^(H_2_O) and F^–^(H_2_O)(2*ν*_IHB_). Note the log scale.

The inference of enhanced rotational excitation in the HF + OH product channels for photodetachment is not supported by the theoretical predictions. In Fig. 11, the rotational state distribution of HF is displayed at several different eKE on the X and A states, respectively. Note that the rotational state distribution of OH is not shown as it is the same as that of HF because of the *J* = 0 constraint. All HF rotational state distributions indicate limited excitation in the HF product and this is true at all eKE values on both the X and A states. The strongest *j*_HF_ excitation takes place at eKE = 0.20 eV (brown lines). The maximum rotational angular momentum quantum numbers of HF are close to 12 in all the panels, and the corresponding rotational energy in the HF + OH products is about 0.2 eV. The relatively small rotational excitation in both HF and OH is consistent with previous experimental^26–28^ and theoretical results^4,30,32,57^ examining the dynamics of F + H_2_O reaction as promoted by bimolecular collisions. It is interesting to note, however, that also in the ground state results for FH_2_O reported in ref. 13 one of the most significant differences between theory and experiment was a broad region of rotationally excited products in the (0,0) manifold. The eKE signature for these events was also shown in ref. 13 to correspond to a broad product channel Feshbach resonance as seen in the ‘stable’ photoelectron spectrum surviving after 2 ps wavepacket propagation. It is possible that some of the discrepancies in apparent rotational excitation seen in the present work likewise derive from the dissociation dynamics of long-lived resonance states that are not effectively captured in short wavepacket propagations, and the interactions of these states with the bending potential on the neutral surface. In addition, as noted earlier, IVR into states with significant bending excitation may also play a role given the fact that these PPC measurements are integrated over nearly 50 ms trapping times following IR excitation.

The role played by OH vibrational excitation is also worth mentioning. In the theoretical predictions for both F^–^(H_2_O) and F^–^(H_2_O)(2*ν*_IHB_), vibrational excitation of OH is very low and actually reduced for F^–^(H_2_O)(2*ν*_IHB_). In the experimental results in ref. 13 however, a clear feature at the threshold for (0, 1) OH-excited products was observed, with the full range of product KER, implying involvement of both the X and A states. This is also seen in the no-IR PPC spectra reported in Fig. 4(b). However, the difference spectra, particularly the *E*_TOT_ spectrum in Fig. 5, shows that in accord with the theoretical predictions there is no enhancement of the (0, 1) channel with vibrational excitation. From a theoretical perspective, in general, the HF vibrational state distribution is inverted when excited state product channels open, while little vibrational excitation is found for the OH product. This trend is consistent with both experimental studies of full collisions^26–28^ and theoretical observations^4,30,32,57^ in the F + H_2_O bimolecular reaction. The experimentally observed (0, 1) products implies that there are likely some details of the anionic or neutral potential energy surfaces that need further examination. This includes the excited A state, since the large range of KERs observed at the energetic threshold for (0, 1) is consistent with direct dissociation involving repulsive regions of both the X and A states.

## 6. Conclusions

This study reports a joint experiment-theory examination of the effects of overtone excitation of the ionic hydrogen bond, F^–^(H_2_O)(2*ν*_IHB_) on the dissociative photodetachment and thus the impact of parent anion vibrational excitation on the dynamics of the F + H_2_O → HF + OH reaction. Comparing the experimental and theoretical PPC difference spectra, the key features of enhancement and depletion are reproduced by theory quite well. These results offer insights into dynamics induced by photodetachment in a large and different Franck–Condon region on the neutral PES. There are, however, several outstanding differences between theory and experiment for both F^–^(H_2_O) and F^–^(H_2_O)(2*ν*_IHB_). These include the role of rotational excitation in the HF + OH product channels and the level of OH vibrational excitation induced in the products. Extension of theoretical treatments to the decay of long-lived resonance states would be of great interest in resolving this question, as well as explicitly accounting for IVR prior to photodetachment. An improved A state PES is another area that deserves examination.^31^ In addition, at some level an explicit account for the photodetachment dynamics, beyond the s-wave approximation and taking into account the Wigner threshold law that governs the energy dependence of photodetachment cross sections will become important.^58^ Considerable improvements in the experiment would also be worthwhile, in particular increasing the excitation fraction for F^–^(H_2_O)(2*ν*_IHB_) and an improvement in the eKE spectral resolution to the level achieved by Neumark and co-workers using the slow electron velocity map imaging (SEVI) technique.^59^ With sufficient resolution and signal-to-noise, the time-dependent eKE could provide a direct experimental probe of IVR in the excited F^–^(H_2_O) anion. SEVI-level resolution has yet to be achieved in three-dimensional coincidence photoelectron imaging measurements, however. Experiments with colder parent anions using a new cryogenically cooled octupole accumulator trap would also be useful in better understanding the role of parent anion rotational excitation on the observed dynamics.^60^ In summary, the present joint experiment-theory study of the effects of vibrational excitation on the F + H_2_O → HF + OH reaction has been an extensive undertaking at the ‘benchmarking Frontier’ for four atom reactions, and illustrates the state-of-the-art for full-dimensionality quantum dynamics of four atom reactions.

## Conflicts of interest

There are no conflicts of interest to declare.
