# Supplementary material for: Effects of vibrational excitation on the F + H2O → HF + OH reaction: dissociative photodetachment of overtone-excited [F–H–OH]–
Source: Chem Sci. 2017 Sep 25;8(11):7821–33. doi: 10.1039/c7sc03364h (PMC5674243; doi:10.1039/c7sc03364h)
Supplement: Supplementary file 1 [file SC-008-C7SC03364H-s001.pdf]

Electronic Supplementary Material (ESI) for Chemical Science.
This journal is © The Royal Society of Chemistry 2017

Effects of Vibrational Excitation on the F + H_2O → HF + OH Reaction: Dissociative Photodetachment of Overtone Excited [F–H–OH]$^-$

Amelia W. Ray,[1] Jianyi Ma,[2,*] Rico Otto,[1,†] Jun Li,[2] Hua Guo,[3] and Robert E. Continetti[1,*]

[1]*Department of Chemistry and Biochemistry, University of California, San Diego, 9500 Gilman Drive, La Jolla, California 92093-0340, USA*
[2]*Institute of Atomic and Molecular Physics, Sichuan University, Chengdu, Sichuan 610065, China*
[3]*School of Chemistry and Chemical Engineering, Chongqing University, Chongqing 401331, China*
[4]*Department of Chemistry and Chemical Biology, University of New Mexico, Albuquerque, New Mexico 87131, USA*

Electronic Supplementary Information

I. Theory

The coordinates used in the wavepacket calculations are shown in **Figure S1**. The parameters of the numerical calculations are shown in **Table S1**.

In **Figures S2** and **S3**, the calculated HF and OH vibrational state distributions on the X/A state PES are given in several energies for the vibrational ground and excited states of the anion, respectively. In both cases, the HF vibrational state distribution is inverted when excited states are energetically accessible, while little vibrational excitation is found for the OH product. **Figure S2** shows that parent anion vibrational excitation leads to larger populations in both the HF(v=0) and HF(v=2) states. This trend is consistent with both experimental[1-3] and theoretical observations[4-7] in the F + H_2O bimolecular reaction, and is a level of detail that cannot be extracted from the experimental PPC spectrum.

In **Figure S4**, the total photoelectron spectrum is compared with the dissociation flux after the ~1 ps propagation. This shows that that the dissociation captured by the propagation is disproportionally from high energy resonances. In other words, there remains a significant population of lower energy longer-lived resonances that dissociate on a timescale beyond 1 ps.

In **Figure S5**, the calculated internal energy distributions of the HF + OH and F + H_2O channels are shown at two energies for both the vibrational ground and excited state of the anion.

II. Experiment

The energetics of various channels are summarized in **Table S2**. **Figure S6** shows PPC difference spectra at IR photon energies of 2885, 2872 and 2900 cm^{-1}, and a null difference spectrum, to provide a measure of the magnitude of the effect. The 2885 cm^{-1} spectrum exhibits the strongest effect of vibrational excitation, although the 2872 cm^{-1} spectrum also shows statistically significant enhancement above a total energy of 1.0 eV. A measure of the statistical error in the 2885 cm^{-1} difference spectrum can also be examined in **Figure S7**, which shows the no-IR PPC spectrum as the number of events N(eKE,KER) in frame (a) and the Poisson error $N(eKE,KER)^{1/2}$ in frame (b).

II.1. Stable Photoelectron Spectra and Estimation of Fraction of Vibrational Excitation

The photoelectron spectra for stable products, events that lead to the detection of one photoelectron and a single particle at the center-of-mass of the incident ion beam, are measured in these experiments as well. In the previous study of cold $F^-(H_2O)$ anions in ref. 8, a product-channel complex was observed above the KE_{MAX}^{UV} limit (1.03 eV) as well as long-lived vibrational Feshbach resonances near eKE = 0.4 eV and 0.0 eV. Given the finite resolution of the neutral-particle detector, dissociative events with very small KER cannot be directly distinguished from true stable events where a single particle arrives at the detector following the 7μs flight time.

Figure S8 shows a difference spectrum of the effect of anion vibrational excitation on the stable photoelectron spectrum, as well as a difference spectrum for the dissociative photoelectron spectrum. The stable spectrum is dominated by suppression while the dissociative spectrum is dominated by enhancement, consistent with a decrease in the production of stable complexes when $2\nu_{IHB}$ is excited in $F^-(H_2O)$. For reference, the energetic limits, both IR-excited and no-IR, for dissociation to HF + OH and F + H_2O channels are indicated on each difference spectrum by solid and dashed vertical lines, respectively. Focusing on the stable component for the moment, clear enhancement regions are evident near eKE = 0.2, 0.6, and >1.2 eV. The highest-energy feature straddling the KE_{MAX}^{UV+IR} is the

stable product-channel complex previously observed in the no-IR experiments reported in Ref. 8, shifted to higher eKE by the IR photon energy. This shows that there are events where the IR photon energy stored as vibrational energy in the precursor anion has produced a classic 'hot band' in the photoelectron spectrum, producing vibrationally cold ground-state exit channel FH-OH H-bonded complexes and higher eKE electrons. Similar to the no-IR case, the stable product-channel FH-OH complex appears as two enhancement peaks straddling KE_{MAX}^{UV+IR}, shown in the stable-channel difference spectrum in the upper panel of **Figure S8**. The higher energy enhancement peak appears at eKE = 1.6 eV, or roughly 0.2 eV higher in energy than KE_{MAX}^{UV+IR}, consistent with the no-IR stable photoelectron spectrum reported in ref. 8. An estimate of the fraction of the precursor-ion packet excited by IR-laser irradiation can be determined by comparing the signal for the product channel complex in the difference spectrum to the no-IR stable eKE spectrum. As shown in **Figure S9**, a model was used where the excited spectrum was estimated using a combination of the no-IR spectrum and the no-IR spectrum shifted by the IR photon energy and scaled by the excitation fraction, *f*, such that $IR_{model} = f(IR) + (1-f)$*no-IR). A simulated difference spectrum was then taken by subtracting the no-IR spectrum from IR_{model} using the relationship $\Delta_{model} = IR_{model}$ –no-IR. This model difference spectrum was then compared to the experimental difference spectrum in the region corresponding to the stable product-channel complex (above 1.0 eV). The excitation factor *f* was determined by scaling until integrated signal in the model and experimental difference spectra were the same. Using this model, ~ 4% of the anion packet is determined to excited for the $h\nu_{IR}$ = 2885 cm^{-1} data shown here. However, this determination assumes that Franck-Condon overlap of excited anions with stable product-channel complex is unchanged and that additional energy in the IR-excited systems is partitioned into photoelectron eKE, and therefore this excitation fraction should be treated as a best estimate only.

Supplementary Material References

1. M. Ziemkiewicz and D. J. Nesbitt, *J. Chem. Phys.*, 2009, **131**, 054309.
2. M. Ziemkiewicz, M. Wojcik and D. J. Nesbitt, *J. Chem. Phys.*, 2005, **123**, 224307.
3. A. M. Zolot and D. J. Nesbitt, *J. Chem. Phys.*, 2008, **129**, 184305-184309.
4. J. Li, R. Dawes and H. Guo, *J. Chem. Phys.*, 2012, **137**, 094304.

5. J. Li and H. Guo, *Chin. J. Chem. Phys.*, 2013, **26**, 627-634.
6. J. Li, B. Jiang and H. Guo, *Chem. Sci.*, 2013, **4**, 629-632.
7. B. Zhao and H. Guo, *J. Phys. Chem. Lett.*, 2015, **6**, 676-680.
8. R. Otto, J. Ma, A. W. Ray, J. S. Daluz, J. Li, H. Guo and R. E. Continetti, *Science*, 2014, **343**, 396-399.
9. P. Weis, P. R. Kemper, M. T. Bowers and S. S. Xantheas, *J. Am. Chem. Soc.*, 1999, **121**, 3531-3532.
10. C. Blondel, P. Cacciani, C. Delsart and R. Trainham, *Phys. Rev. A*, 1989, **40**, 3698.
11. B. Ruscic, Active Thermochemical Tables (ATcT) version 1.118, http://ATcT.anl.gov, (accessed April 2017).

Table S1. Numerical parameters (in a.u.) used in wave packet calculations. The HF+OH channel is described by diatom-diatom Jacobi coordinates and the F+H_2O channel is described by (2+1) Radau-Jacobi coordinates.

Channels	HF+OH	F+H_2O
Grid/basis ranges and sizes	$r_0 \in (2.0, 17.0)$ $N_0 = 135$	$r_0 \in (3.0, 13.0)$ $N_0 = 140$
	when $1 \le i_0 \le 60$,<br>$r_1 \in (0.9, 5.6), N_1 = 28$	when $1 \le i_0 \le 60$,<br>$r_1 \in (1.3, 4.1), N_1 = 20$
	when $61 \le i_0 \le 140$,<br>5 vibrational basis for r_1	when $66 \le i_0 \le 140$,<br>5 vibrational basis for r_1 and r_2
	5 PODVR for r_2	
Largest values of j_1, j_2 and m	37, 32, 32	24, 24, 24
Damping potential for r_0 [a]	$\alpha_{abs} = 0.08$, $r_{0,abs} = 13.5$	$\alpha_{abs} = 0.08$, $r_{0,abs} = 10.3$
Damping potential for r_1 [a]	$\alpha_{abs} = 0.08$, $r_{i,abs} = 3.6$	$\alpha_{abs} = 0.08$, $r_{i,abs} = 2.7$
Position of the product state	$r_{0,p} = 13.0$	$r_{0,p} = 10.0$
Propagation steps for X state	25000	20000
Propagation steps for A state	25000	20000

[a]The damping function is defined as $D = \exp[-\alpha_{abs}(r - r_{abs})^2]$, $r \ge r_{abs}$

Table S2. Experimentally determined total kinetic energy (eKE + KER) limits for accessible product channels for both UV-only and UV+IR DPD of $F^-(H_2O)$. The maximum kinetic energies are reported for neutral products formed in their ground rotational and vibrational states. Following Otto *et al.* [8], KE'_{MAX} for production of F + H_2O + e- in the DPD of $F^-(H_2O)$ is determined from the measured dissociation energy $F^-(H_2O) \rightarrow F^- + H_2O$ (ΔD^o = 1.14 eV [9], the photon energy ($h\nu_{UV}$= 4.80 eV) and the electron affinity of the F atom (3.401 eV)[10]. KE_{MAX} for production of HF(n_{HF}=0) + OH(n_{OH}=0) is determined by the reaction exoergicity of -0.76 eV based on heat of formation data in the Active Thermochemical Tables.[11] The product channel, HF + OH, is labeled using the notation (n_{HF}, n_{OH}) to indicate quanta of vibrational excitation. The UV+IR kinetic energy limits are found by adding the IR photon energy ($h\nu_{IR}$= 0.36 eV, 2885 cm^{-1}) to $h\nu_{UV}$, under the assumption that the added IR photon energy may appear as product or photoelectron kinetic energy.

**Product Channel**	**Kinetic Energy Limit UV (eV)**	**Kinetic Energy Limit UV + IR (2885 cm^{-1}) (eV)**
Product KE_{MAX} (HF + OH): (0,0)	1.03	1.39
(0,1)	0.58	0.94
(1,0)	0.54	0.89
(1,1)	0.09	0.45
(0,2)	0.16	0.52
(2,0)	0.07	0.43
(0,3)	--	0.12
Reactant KE'_{MAX} (F + H_2O)	0.26	0.62

(a)

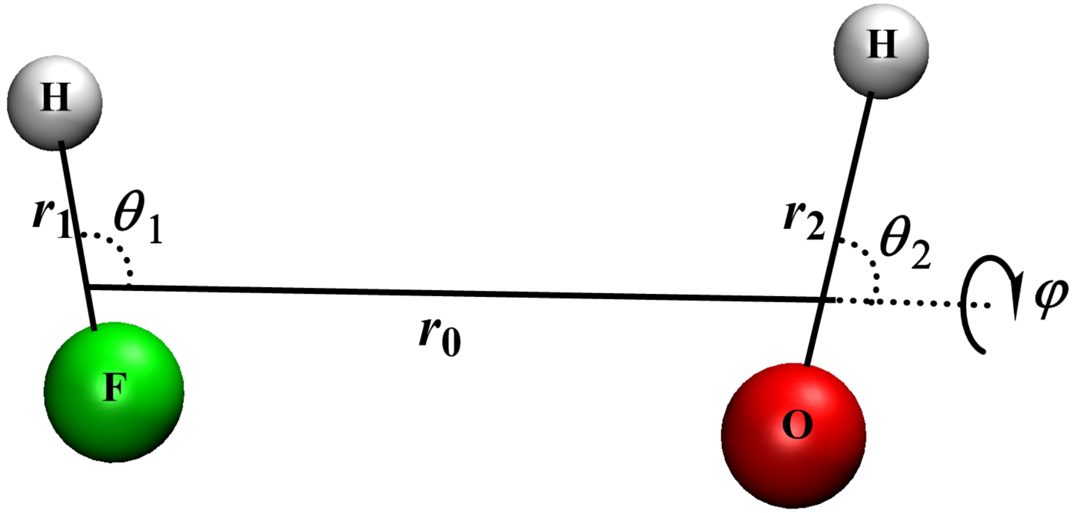

(b)

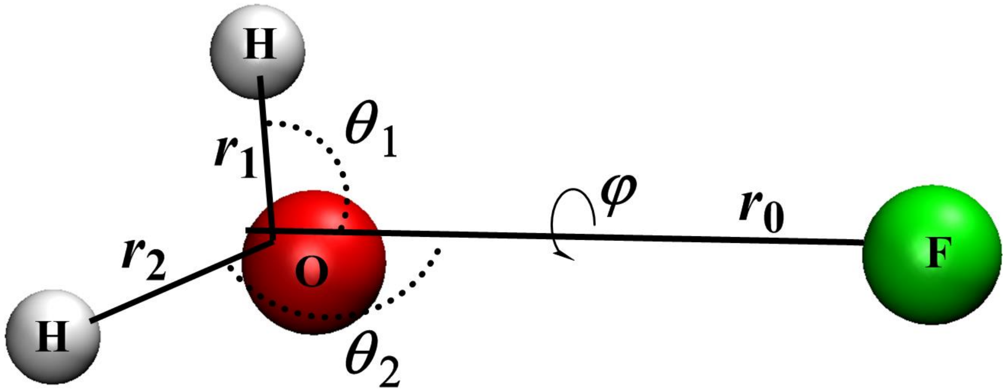

Figure S1. Diatom-diatom Jacobi coordinates (a) for the HF + OH channel and (2+1) Radau-Jacobi coordinates (b) for the F + H_2O channel.

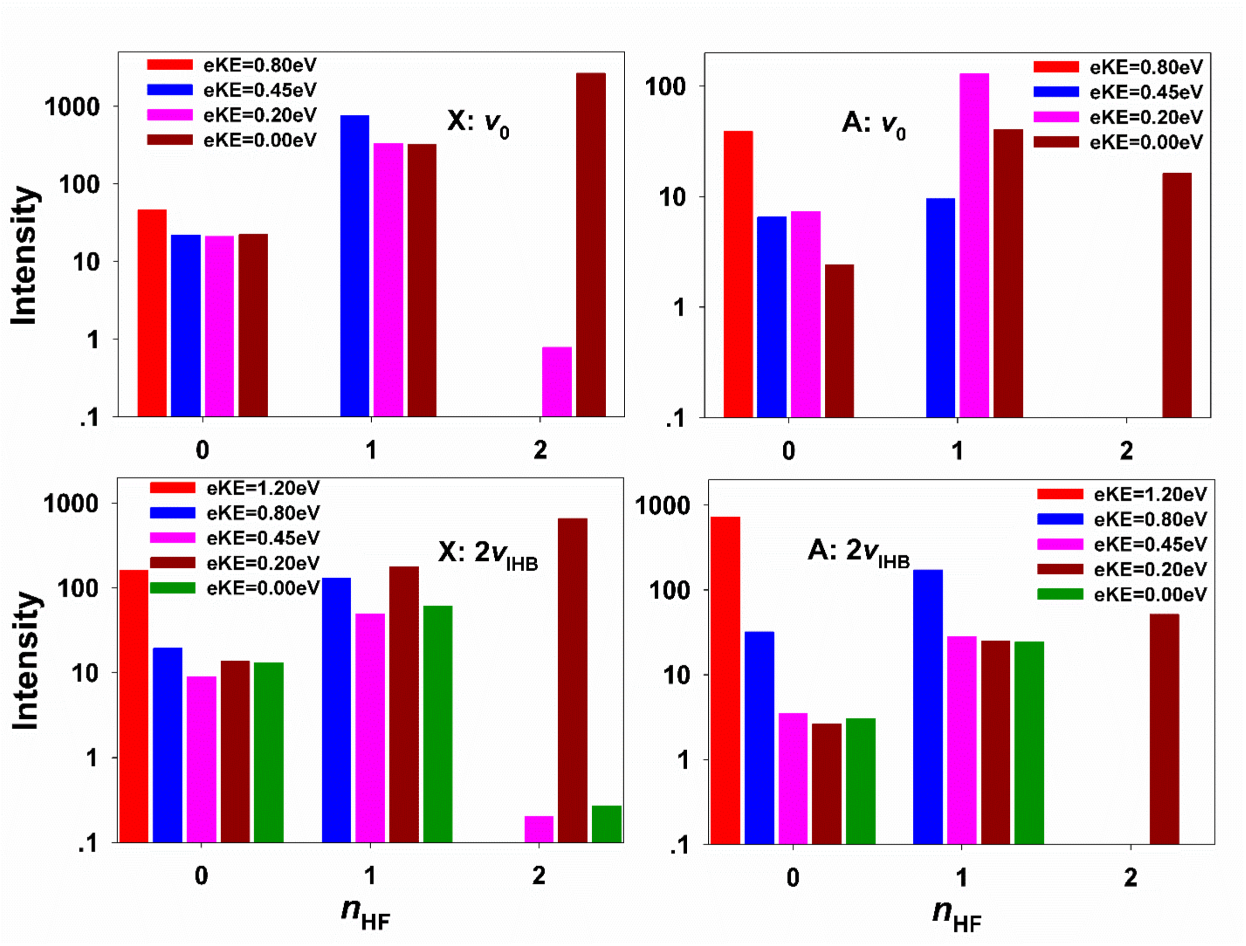

Figure S2. The HF(n_{HF}) vibrational state distributions for the HF+OH channel on the *X*/*A* state PESs at several different eKEs for the ground and vibrational excited anion. Note the log scale.

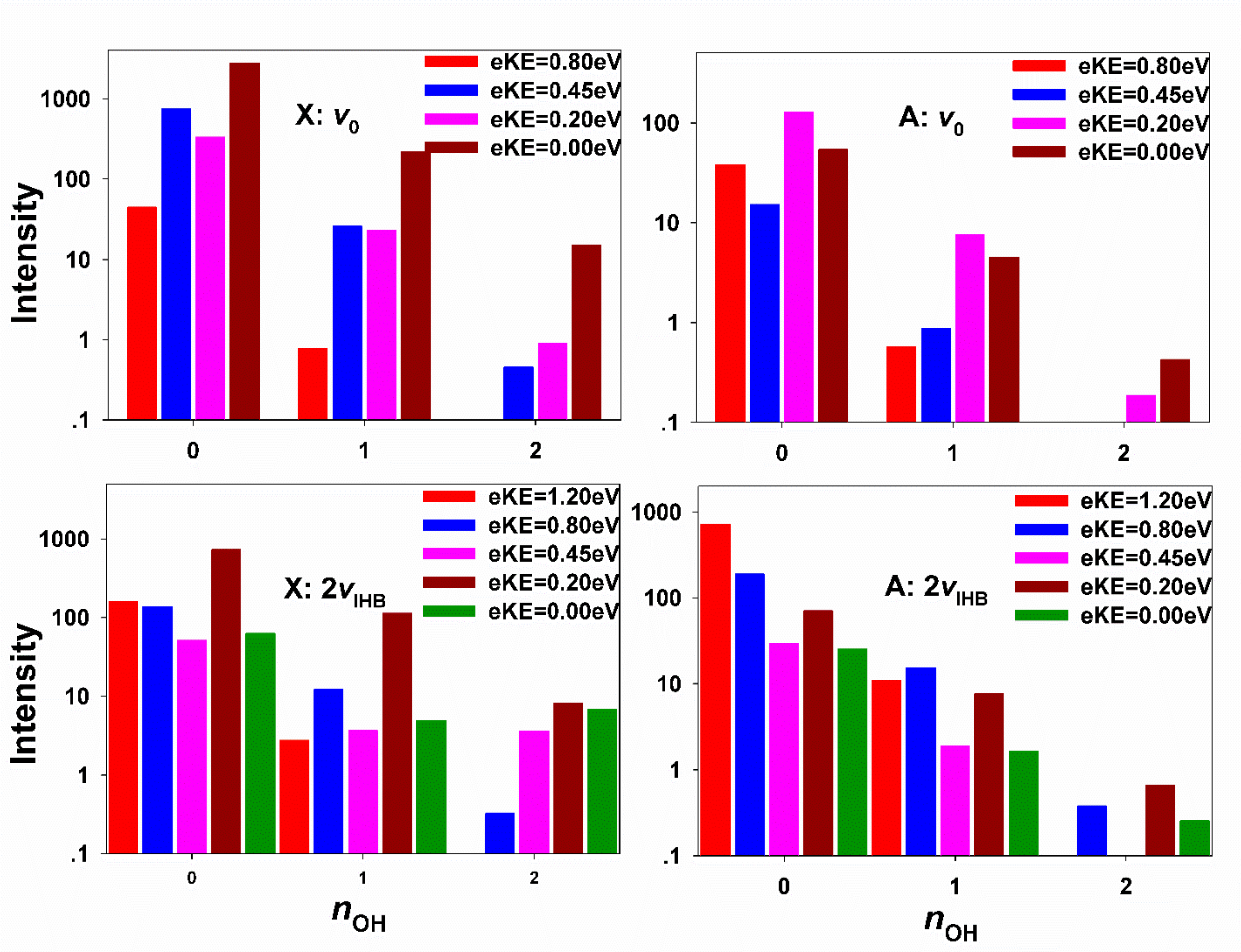

Figure S3. The OH(n_{OH}) vibrational state distributions for the HF+OH channel on the X/A state PESs at several different eKEs for the ground and vibrational excited anion. Note the log scale.

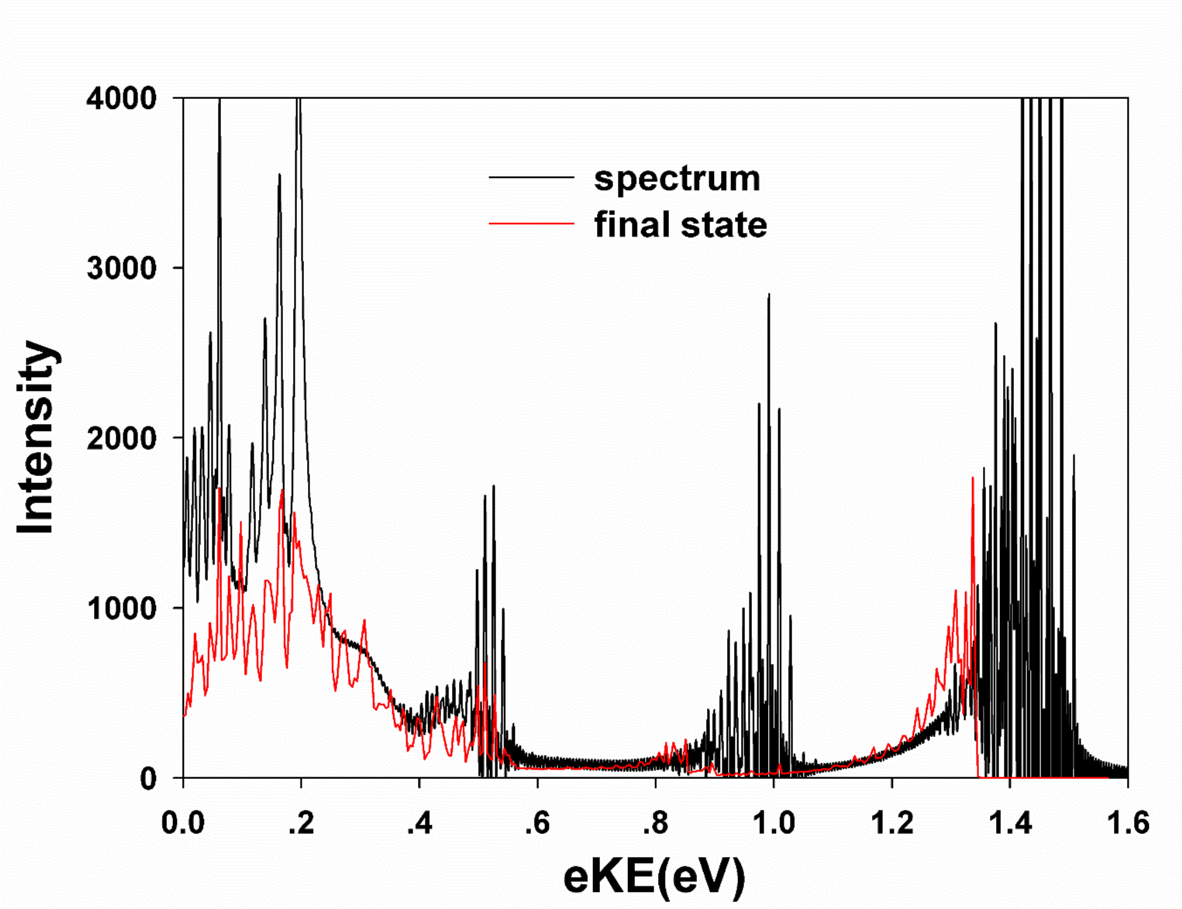

Figure S4. Outgoing flux and the photoelectron spectrum for photodetachment of the vibrationally excited $F^-(H_2O)$anion onto the X state of FH_2O. The red line indicates the flux captured in a 1ps propagation, while the black line is the total spectrum.

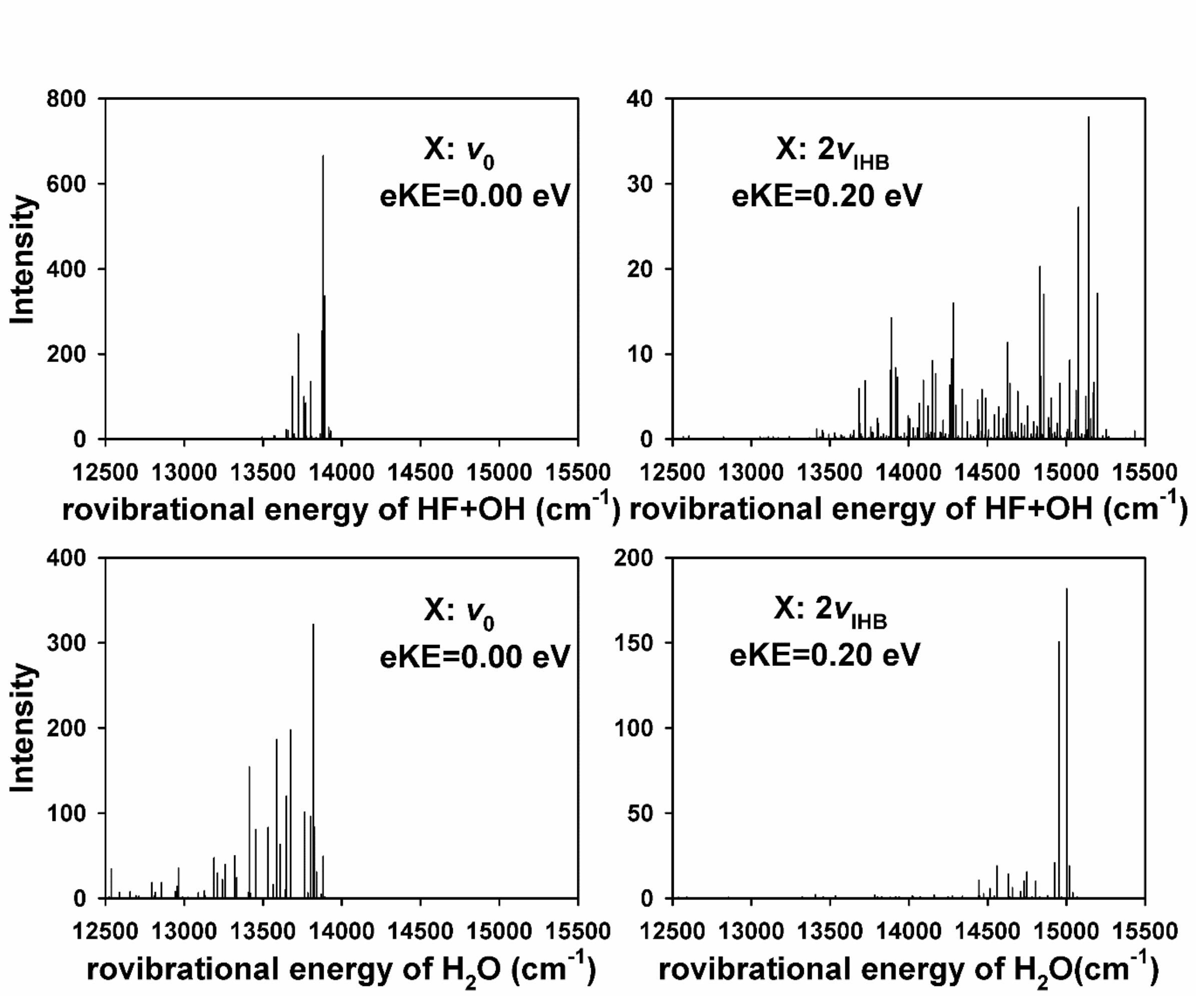

Figure S5. Product H_2O internal energy distributions in the F + H_2O channel at the total energy of 1.72 eV (eKE=0.0 eV) for the ground anion and 1.87 eV (eKE=0.20 eV) for the vibrationally excited anion.

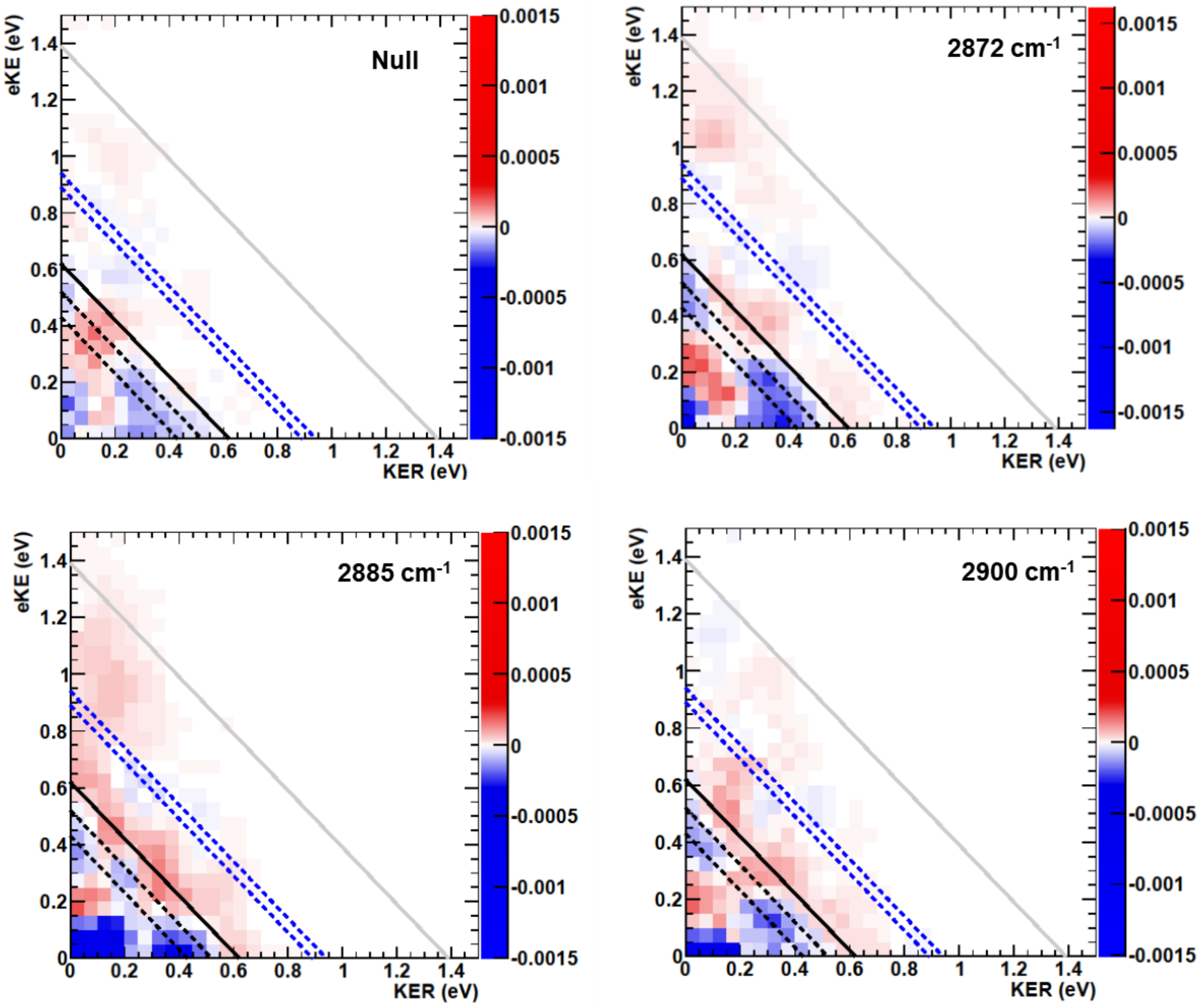

Figure S6. Difference (IR – no-IR) PPC spectra recorded with $h\nu_{UV}$= 4.80 eV at various IR photon energies, and a null difference PPC spectrum for $F^{-}(H_2O)$ dissociative photodetachment at top left. Spectra have been normalized to the number of events in the no-IR spectrum (or in the case of the null spectrum, the subtracted spectrum) to put them all on a common scale to see the relative effects. The grey and black solid lines indicate the energetic limits, KE_{MAX}, for dissociation into HF + OH and F + H_2O fragments, respectively, determined by the total photon energy $h\nu_{UV}$ + $h\nu_{IR}$. The dashed lines indicate vibrationally excited product states as in the other PPC spectra. The blue areas indicate suppression and the red enhancement relative to the no-IR spectrum. In particular by examining the region above 0.6 eV, it can be seen that the 2885 cm^{-1} spectrum has the most significant signal, with the 2872 cm^{-1} spectrum also showing significant signal above 1.0 eV.

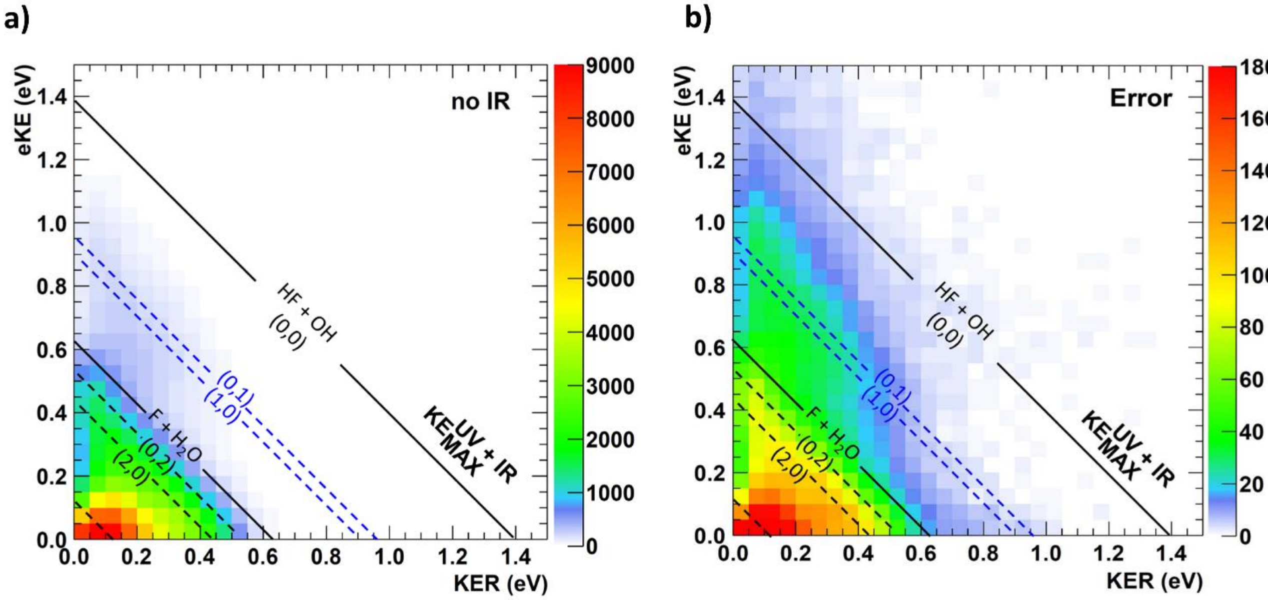

Figure S7. Frame (a) shows the no-IR PPC spectrum with an integer-scaled color bar showing N(eKE,KER), the number of events per (eKE, KER) bin, in the two-dimensional spectrum. Frame (b) shows the Poisson error per bin as $N(eKE,KER)^{1/2}$, to provide a calibration for the statistical significance of the difference plots in Figure 4(a) and Figure S6.

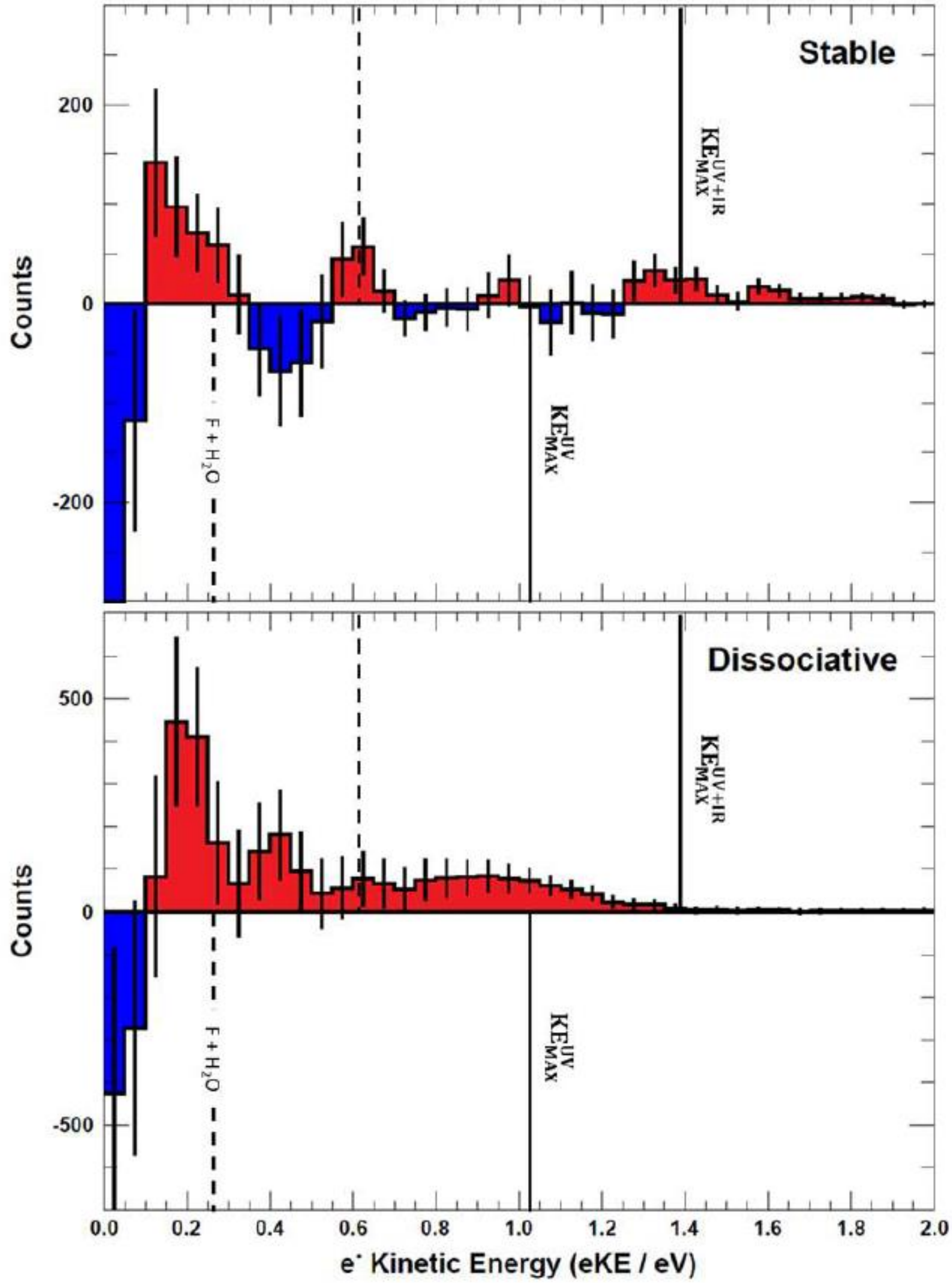

Figure S8. Difference (IR – no-IR) spectra for the stable and dissociative photoelectron spectra, showing IR-excited (top half of each frame) and no-IR (bottom half of each frame) energetic limits. Blue and red areas indicate suppression and enhancement, respectively, relative to the no-IR spectrum. Solid and dashed vertical lines correspond to limits for dissociation to HF + OH and F + H_2O, respectively. Error bars correspond to $\sqrt{N_{IR} + N_{no-IR}}$ for each bin.

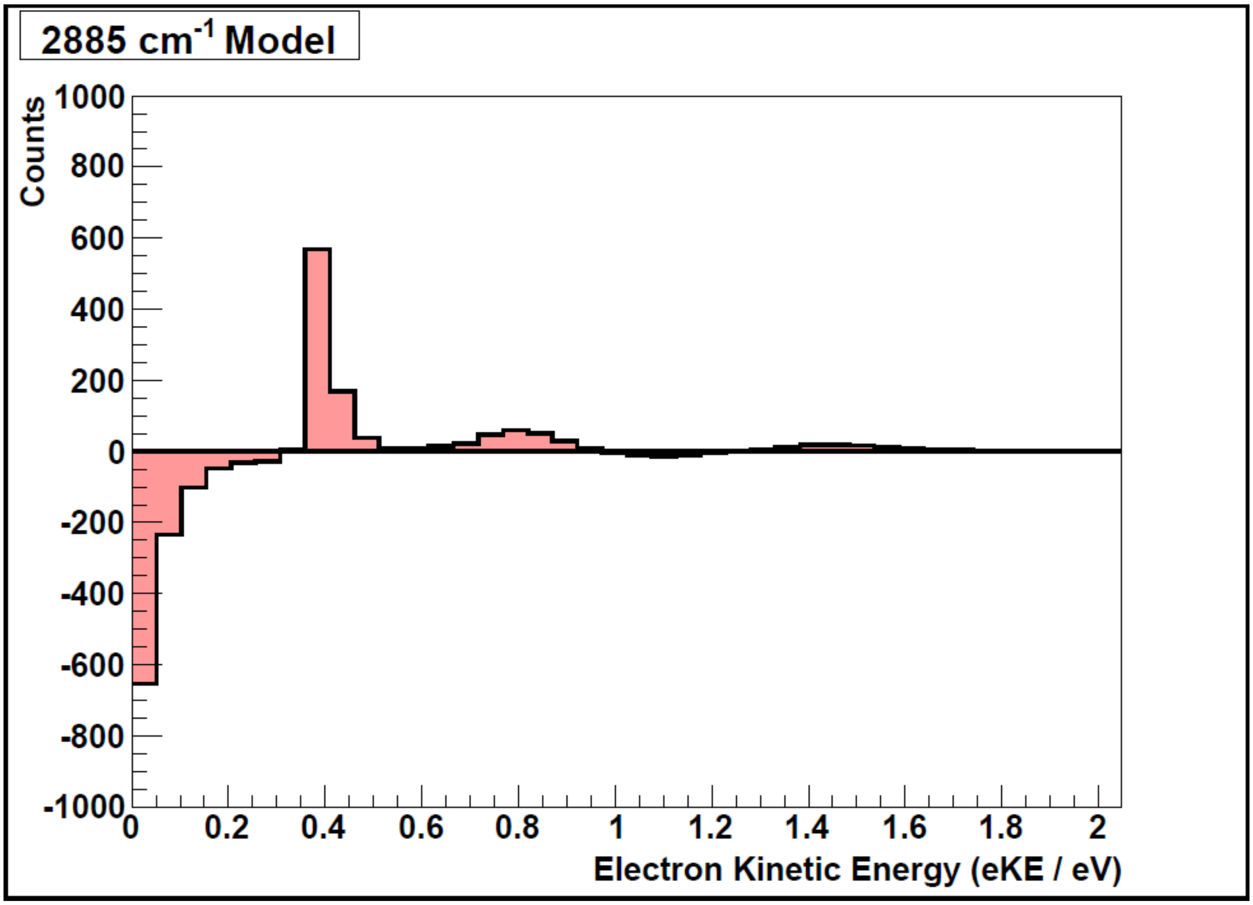

Figure S9. Simulated difference photoelectron spectrum for estimation of the fraction of vibrational excitation with the simple model where the UV + IR photoelectron spectrum was generated by summing the no-IR spectrum with the no-IR spectrum shifted by the IR photon energy as the pure IR contribution, scaled by the excitation fraction, *f*, such that IR_{model} = *f*(IR) + (1-*f*)*(no-IR). The simulated difference spectrum shown here results from subtracting the no-IR spectrum from IR_{model}: Δ_{model}= IR_{model} –no-IR.
